# ﻿The subfamily Attageninae (Coleoptera, Dermestidae) from Saudi Arabia

**DOI:** 10.3897/zookeys.1243.146325

**Published:** 2025-06-25

**Authors:** Jiří Háva, Mahmoud S. Abdel-Dayem, Hathal M. Aldhafer

**Affiliations:** 1 Private Entomological Laboratory and Collection, Rýznerova 37/37, CZ-252 62 Únětice u Prahy, Prague-west, Czech Republic Private Entomological Laboratory and Collection Prague Czech Republic; 2 King Saud University Museum of Arthropods (KSMA), Plant Protection Department, College of Food and Agricultural Sciences, King Saud University, P.O. Box 2460 Riyadh 11451, Saudi Arabia King Saud University Riyadh Saudi Arabia; 3 Entomology Department, Faculty of Science, Cairo University, Giza, 12613, Egypt Cairo University Giza Egypt

**Keywords:** Arabian Peninsula, checklist, distribution, elevation, endemic species, historical records, new records, skin beetles

## Abstract

This study documents the diversity and distribution of the subfamily Attageninae (Coleoptera: Dermestidae) in Saudi Arabia based on the examination of specimens from institutional and private collections, and field surveys using different trapping and collection methods. It enumerates 20 species belonging to two genera, *Attagenus* (19 species) and *Telopes* (one species). Six species are newly recorded for the country: *Attagenusbarbieri* Pic, 1946; *A.jakli* Háva, 2021; *A.kadleci* Háva, 2012; *A.vanharteni* Háva, 2009; *A.yemensis* Háva & Herrmann, 2014; and *Telopestessellatus* Reitter, 1887. Morphological examinations led to the exclusion of five previously misidentified species from the Saudi fauna: *A.dichrous* Roth, 1851; *A.fasciolatus* (Solsky, 1876); *A.heydeni* (Reitter, 1881); *Telopesobtusus* (Gyllenhal in Schönherr, 1808); and *Telopesreitteri* (Mroczkowski, 1968). The distribution of Attageninae reveals the influence of environmental gradients on species richness, with mid-altitude areas (601–1500 m) serving as biodiversity hotspots, hosting 14 species. Lowland and highland specialists display niche adaptation, with species like *A.apicalis* and *A.logunovi* restricted to low altitudes (≤ 600 m) and *A.kadleci* found exclusively in high-altitude environments (> 2000 m). Approximately 40% of the Attageninae species in Saudi Arabia are found within protected areas. This study identifies five endemic species in the Arabian Peninsula, including one exclusive to Saudi Arabia (*A.logunovi*). These findings increase the number of known attagenine species by 43% in Saudi Arabia and 7% in the Arabian Peninsula, highlighting the need for systematic surveys and taxonomic revisions to reveal the overlooked biodiversity in the region.

## ﻿Introduction

The subfamily Attageninae (Coleoptera: Dermestidae) is a widely distributed group of skin beetles, with the majority of species occurring in the Palaearctic, Afrotropical, and Nearctic regions (Háva 2015b, 2020, 2024). Globally, the subfamily comprises five tribes, 15 genera, and approximately 311 species (Háva 2024). The Palaearctic region hosts approximately five genera with 75 species, while the Afrotropical, Nearctic, Neotropical, and Oriental regions harbor seven genera with 45 species and four with 28 species, respectively (Háva 2015b, 2024).

Members of Attageninae are small (2.0–9.0 mm), oval-shaped beetles with distinctive scales or bristles and mottled coloration. Ecologically, larvae of many species play critical roles as decomposers by feeding on keratinous and decaying organic materials, including animal remains (Kadej et al. 2020). Adult beetles occasionally contribute to pollination (Muñoz-Rodríguez et al. 2024), but are more commonly associated with their pest status, causing economic damage to textiles, stored products, and museum collections (Hinton 1945; Panagiotakopulu 2003; Hagstrum and Subramanyam 2009; El-Hassan et al. 2021).

Among Dermestidae, *Attagenus* Latreille, 1802, is the type genus of Attageninae and is the most diverse within the subfamily, with ~ 240 species (Háva 2015b, 2024). Recent taxonomic revisions have elevated the subgenera *Aethriostoma* Motschulsky, 1858 (8 species) and *Telopes* Redtenbacher in Russegger, 1843 (18 species) to independent separate genera (Zhou et al. 2022). Despite this global diversity, the Attageninae fauna of Saudi Arabia remains poorly studied. Currently, only two genera, *Attagenus* and *Telopes*, have been recorded in the country (Háva 2024). Early studies (Shalaby 1961; Mroczkowski 1979, 2002; Zhantiev 2005; Háva 2007, 2011, 2015a; Abdel-Dayem and Háva 2020) provided limited records of Attageninae in Saudi Arabia, highlighting significant gaps in knowledge. This paper represents the third study on the family Dermestidae in Saudi Arabia, following earlier works on the subfamily Thorictinae (Háva et al. 2021) and the subfamily Dermestinae (Háva et al. 2023).

This study aims to fill gaps in faunal records by documenting new records, revising misidentified species, and providing an updated checklist of Attageninae in Saudi Arabia. The findings will enhance our understanding of Attageninae and its biodiversity within the biogeographically vital Arabian Peninsula in regards to our knowledge of this group of beetles.

## ﻿Materials and methods

### ﻿Specimen sources and collection

This study is primarily based on specimens curated at the King Saud University Museum of Arthropods (**KSMA**) in Riyadh, Saudi Arabia, as the main source of faunal data and newly recorded species. Additional specimens were examined from international institutional and private entomological collections (see acronyms below). The collection data spans 1975 to 2023, covering all months of the year.

Adult beetles were collected using different methods, including beating sheets (BS), handpicking (HP), light traps (LT), Malaise traps (ML), pheromone traps (PH), pitfall traps (PT), sticky traps (ST), sweeping nets (SW), and vacuum sampling (VC). These collection efforts covered a wide range of habitats and elevations across different provinces of Saudi Arabia.

### ﻿Depository acronyms

The following acronyms are used in the text to represent depositories of the examined materials:

**FSCA**Florida State Collection and Arthropods, Gainesville, U.S.A.;

**JHAC** Jiří Háva, Private Entomological Laboratory and Collection, Únětice u Prahy, Prague-West, Czech Republic;

**KSMA** King Saud University Museum of Arthropods, Plant Protection Department, College of Food and Agricultural Sciences, King Saud University, Riyadh, Saudi Arabia;

**MMUE**Manchester Museum, The University of Manchester, Manchester, United Kingdom;

**NHMB**Natural History Museum of Basel, Basel, Switzerland;

**NMWC**Natural Sciences National Museum Wales, Cardiff, United Kingdom.

### ﻿Distribution data and nomenclature

Species distributions were visualized using ArcGIS Pro 3.2 to map their Saudi geographic ranges. The global distribution was sourced from the catalogue of Háva (2015b, 2024). The nomenclature is primarily derived from Motyka et al. (2022), with supplementary updates from Zhou et al. (2022). The genus *Attagenus* is treated sensu stricto, adhering to current taxonomic conventions.

Altitudinal records of Attageninae species were grouped into three elevation categories: low (0–600 m), mid (601–1500 m), and high (> 1500 m). For each species, occurrence counts were summed by altitude group and normalized to show relative proportions. A horizontal stacked bar chart was created to visualize these proportions, with color codes representing altitude bands and total record counts labeled at the end of each bar. Data processing and visualization were done using Python.

## ﻿Results

The present study provides a comprehensive review of the subfamily Attageninae (Coleoptera: Dermestidae) based on examining specimens from different localities across Saudi Arabia. A total of 20 species belonging to two genera, *Attagenus* (19 species) and *Telopes* (one species), were identified (Tables 1, 2). Six species are reported as new records for Saudi Arabia (Table 1): *A.barbieri* Pic, 1946; *A.jakli* Háva, 2021; *A.kadleci* Háva, 2012; *A.vanharteni* Háva, 2009; *A.yemensis* Háva & Herrmann, 2014; and *Telopestessellatus* Reitter, 1887.

**Table 1.** List of Attageninae species recorded from Saudi Arabia.

Species	Saudi Arabia	Continental Saudi Arabia
	Farasan Archipelago	
*Attagenusapicalis* Pic, 1942		*?
*Attagenusaristidis* (Pic, 1894)		*
*Attagenusatripennis* Pic, 1938		ONC
*Attagenusbarbieri* Pic, 1942		NR
*Attagenusbeali* Zhantiev, 2005		*
*Attagenuschakouri* Pic, 1907		*
*Attagenuscyphonoides* Reitter, 1881		*
*Attagenusdichrous* Roth, 1851		X
*Attagenusfasciatus* (Thunberg, 1795)		*
*Attagenusfasciolatus* (Solsky, 1876)		X
*Attagenusheydeni* (Reitter, 1881)		X
*Attagenusjakli* Háva, 2021		NR
*Attagenuskadleci* Háva, 2012		NR
*Attagenuslobatus* Rosenhauer, 1856		*
*Attagenuslogunovi* Háva, 2015		*
*Attagenuslynx* (Mulsant & Rey, 1868)		*?
*Attagenuspellio* (Linnaeus, 1758)		*
*Attagenusposticalis* Fairmaire, 1878	*	*
*Attagenusscalaris* (Pic, 1894)		*
*Attagenussmirnovi* Zhantiev, 1973		*
*Attagenusvanharteni* Háva, 2009		NR
*Attagenusyemensis* Háva & Herrmann, 2014		NR
*Telopesobtusus* (Gyllenhal in Schönherr, 1808)	X	
*Telopesreitteri* (Mroczkowski, 1968)		X
*Telopestessellatus* Reitter, 1887		NR

NR, new record; ONC, occurrence not confirmed; X, excluding from the fauna of Saudi Arabia; *, occurrence of the species; ?, needs revision. Farasan Archipelago is a group of islands in the Red Sea.

**Table 2.** A list of Attageninae species recorded from the Arabian Peninsula.

Species	Kuwait	Oman	Qatar	Saudi Arabia		Yemen		United Arab Emirates
				Continent	Farasan Archipelago	Continent	Socotra Island	
*Attagenusapicalis* Pic, 1942				*?				
*Attagenusaristidis* (Pic, 1894)			*	*				*
*Attagenusatripennis* Pic, 1938				*?		*		
*Attagenusbarbieri* Pic, 1942 = *Attagenusheydeni*: Háva & Herrmann, 2009				*				
*Attagenusbeali* Zhantiev, 2005		*		*				*
*Attagenuschakouri* Pic, 1907				*				
*Attagenuscyphonoides* Reitter, 1881 = *Attagenusalfierii* Pic, 1910				*				
*Attagenusfasciatus* (Thunberg, 1795) = *Attagenusgloriosus* (Fabricius, 1798) = *Attagenuscinnamomeus* Roth, 1851	*	*	*	*		*		*
*Attagenusherrmanni* Háva, 2012						*		
*Attagenusjakli* Háva, 2021		*		*				
*Attagenuskadeji* Háva, 2012						*		
*Attagenuskadleci* Háva, 2012				*		*		
*Attagenuskafkai* Háva, 2021		*						
*Attagenuslobatus* Rosenhauer, 1856	*			*				*
*Attagenuslogunovi* Háva, 2015				*				
*Attagenuslynx* (Mulsant & Rey, 1868)			*	*?				
*Attagenusmaseki* Háva, 2013						*		
*Attagenusomanicus* Zhantiev, 2007		*				*		*
*Attagenusornatus* Háva, 2007							*	
*Attagenuspapei* Háva, 2009		*						*
*Attagenuspellio* (Linnaeus, 1758)		*		*		*		*
*Attagenusposticalis* Fairmaire, 1878	*	*	*	*	*	*		*
*Attagenusscalaris* (Pic, 1894)				*				
*Attagenussmirnovi* Zhantiev, 1973		*		*		*		
*Attagenusvanharteni* Háva, 2009		*		*		*		*
*Attagenusyemensis* Háva & Herrmann, 2014				*		*		
*Telopestessellatus* Reitter, 1887				*				

*, occurrence of the species; ?, needs revision.

The revision of historical records led to the exclusion of five species previously listed for the country (Table 1), including *A.dichrous* Roth, 1851; *A.fasciolatus* (Solsky, 1876); *A.heydeni* (Reitter, 1881); *Telopesobtusus* (Gyllenhal in Schönherr, 1808); and *Telopesreitteri* (Mroczkowski, 1968). These exclusions were based on morphological differences and updated distributional data. Similarly, the potential existence of *A.atripennis* Pic, 1938, *A.pellio* (Linnaeus, 1758), and *A.smirnovi* Zhantiev, 1973 remains uncertain (Table 1).

**Figure 1.**
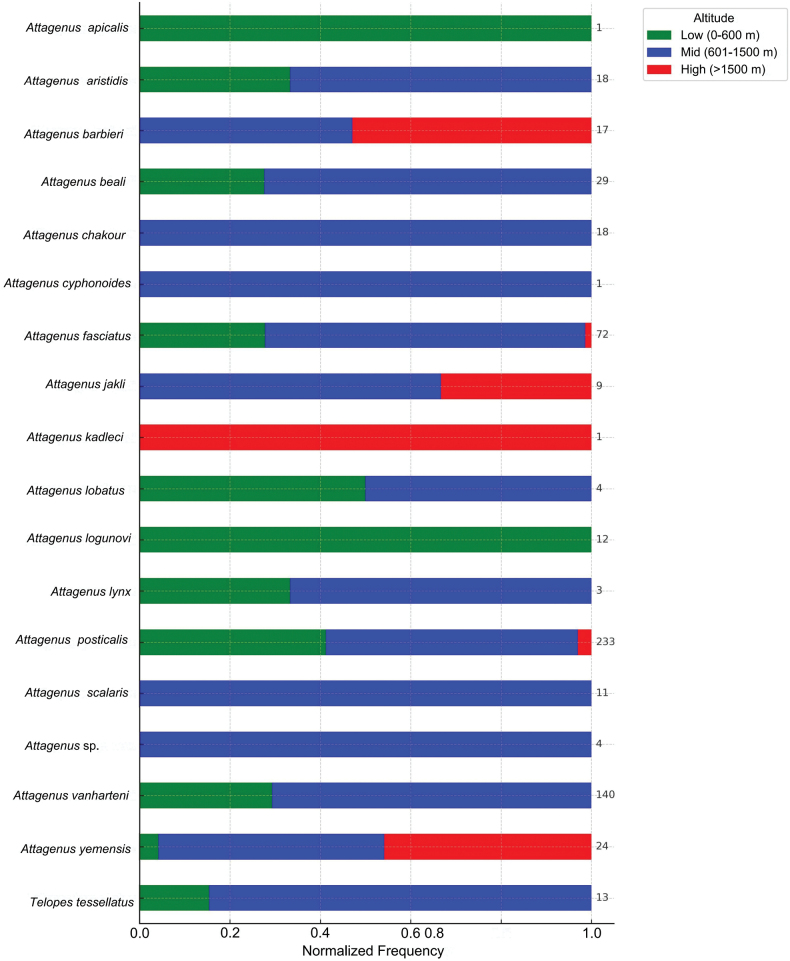
Normalized altitudinal occupancy of Attageninae species in Saudi Arabia. At the end of each bar, a total record count is presented.

The altitudinal distributional patterns of Attageninae species in Saudi Arabia (Fig. 1) demonstrate distinct ecological preferences, with certain species restricted to particular altitudinal ranges, while others exhibit a broader elevational distribution. Species such as *A.apicalis* and *A.logunovi* are restricted to low altitudes (≤ 600 m). In contrast, *A.kadleci* was exclusively found in high-altitude environments (> 2000 m). The mid-altitude range (601–1500 m) supports a diverse assemblage of 14 Attageninae species, including *A.chakouri*, *A.cyphonoides*, and *A.scalaris*. Between lowland (< 500 m) and highland (> 1500 m) habitats, species such as *A.fasciatus*, *A.posticalis*, and *A.yemensis* thrive.

Five of the Attageninae species recorded from the Arabian Peninsula are noteworthy for their possible endemicity. Based on current records, *Attagenusjakli* is confined to Oman and Saudi Arabia; *A.kadleci* is restricted in Saudi Arabia and Yemen; *A.vanharteni* is distributed across Oman, Qatar, Saudi Arabia, the United Arab Emirates, and Yemen; *A.yemensis* is confined to Saudi Arabia and Yemen; and *A.logunovi* is exclusively endemic to Saudi Arabia.

### ﻿Species accounts

**Family Dermestidae Latreille, 1804**

**Subfamily Attageninae Laporte de Castelnau, 1840**

**Tribe Attagenini Laporte de Castelnau, 1840**

**Genus *Attagenus* Latreille, 1802**

#### ﻿ Attagenus apicalis

Taxon classificationAnimaliaColeopteraDermestidae

Pic, 1942

DC2B445E-D2FA-5C2F-BDAF-F3A8863EA913

##### Previous records.

Wadi Shaib Luha (Mroczkowski 1979).

##### Remarks.

Since no additional specimens have been collected since Mroczkowski (1979), the specimen from Wadi Shaib Luha requires further revision.

##### Distribution.

The distribution range of this species is very restricted both locally and globally. Within Saudi Arabia, it is confined to the arid areas of Riyadh Province (Fig. 4) at an elevation of 560 m a.s.l. Globally, its range is limited to Eritrea and Saudi Arabia.

#### ﻿ Attagenus aristidis

Taxon classificationAnimaliaColeopteraDermestidae

(Pic, 1894)

CFC2085A-78EF-5AB0-A5A7-06BE7A87575F

Fig. 2A

##### Previous records.

Riyadh-Dammam (Mroczkowski 1979 as *A.dichrous*) and Eastern Province, Ain Dar (Háva 2011).

##### Material examined.

Saudi Arabia • 2 ex; Riyadh Province, Al Hair, c. 22 km S of Riyadh, W of Al Hair; 24°26.66'N, 46°38.489'E; 14.iii.2023, ing. M. Krejčíř lgt.; JHAC • 1 ex; Riyadh Province, Muzahimiyah, Al Khararah; 24°24'21"N, 46°14'40"E; 30.iii.2011; Y. Drayhim, H. Al Dhafer, A. Al Gharbawy, H. Setyaningrum & A. Al Ansi leg.; SW; J. Háva det.; KSMA • 4 ex; 23 km S of Jaww, route 30; 24°17'1"N, 46°2'49"E; 13–14.iii.2023; M. Šárovec lgt.; JHAC • 4 ex; same collection data as preceding; cca 70 km WSW of Riyadh, 3 km along the route Nr.30; 24°17.172'N, 46°2.014'E; 16–18.iii.2023; ing M. Krejčíř lgt.; JHAC • 3 ex; Riyadh Province, Riyadh-Dammam, km 85; 26.iv.1976; W. Wittmer & W. Büttiker lgt.; *Attagenusdichrous* Mroczkowski det., J. Háva det.; NHMB • 1 ex; Tabuk Province, Al Uyaynah, Abdullah Khader Al Atawi Farm (F118); 28°53.209'N, 36°7.201'E; Alt 723 m; 24.v.2018; H. Al Dhafer et al. leg.; SW; J. Háva det.; KSMA.

**Figure 2.**
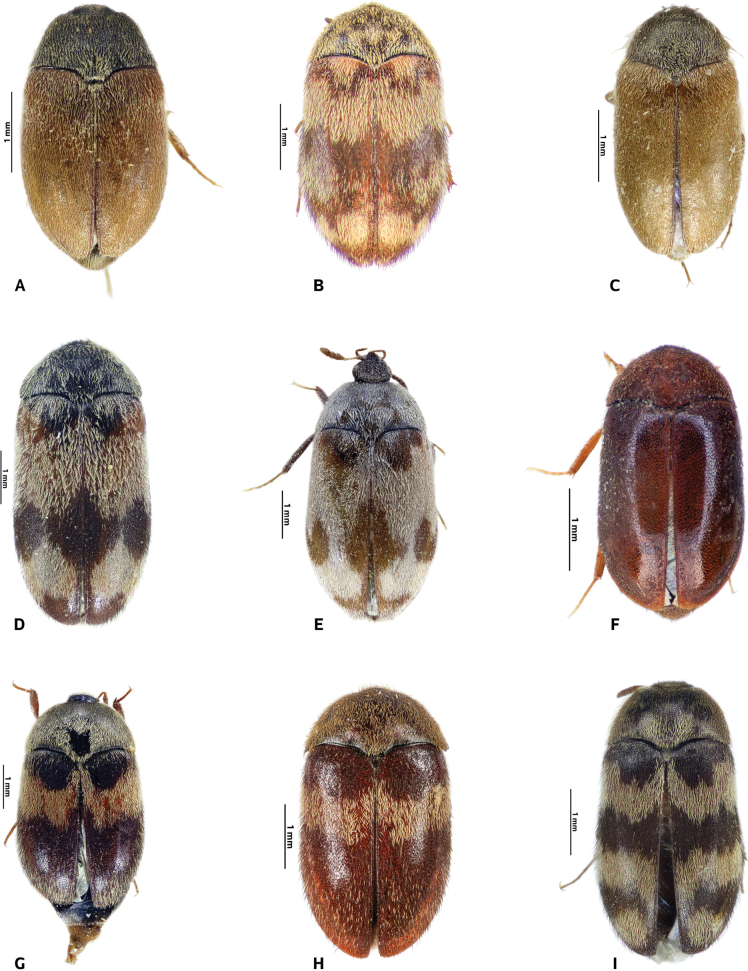
Dorsal habitus of *Attagenus* species **A***A.aristidis* (Pic, 1894) **B***A.barbieri* Pic, 1942 **C***A.beali* Zhantiev, 2005 **D***A.chakouri* Pic, 1907 male **E***A. A.chakouri* Pic, 1907 female **F***A.cyphonoides* Reitter, 1881 female **G, H***A.fasciatus* (Thunberg, 1795) **I***A.jakli* Háva, 2021.

##### Distribution.

This species is distributed in the central (Riyadh Province), eastern (Eastern Province), and northwestern (Tabouk Province) regions of Saudi Arabia (Fig. 4) at elevations ranging from 12 to 746 m a.s.l. The species is distributed across a broad geographical area, spanning from North Africa, including Algeria, Egypt, Libya, Morocco, and Tunisia, extending eastward to the Levant, the Arabian Peninsula, including Qatar, Saudi Arabia, and the United Arab Emirates, and Iran.

#### ﻿ Attagenus atripennis

Taxon classificationAnimaliaColeopteraDermestidae

Pic, 1938

BDDC52FE-5503-50B1-AF22-CDC6ADF3F17C

##### Remarks.

The species was initially described from Yemen, and Mroczkowski (1979) suggested its potential occurrence in Saudi Arabia.

#### ﻿ Attagenus barbieri

Taxon classificationAnimaliaColeopteraDermestidae

Pic, 1946

329DF765-5BF5-5169-8991-DF81162D4F46

Fig. 2B

##### Previous records.

Asir Provence, Abha (Háva and Herrmann 2009 as *A.heydeni*).

##### Material examined.

Saudi Arabia • 1 ex; Asir Province, Abha, Raydah, 18°13.347'N, 42°24.133'E; 2717 m; 26.iv.2014; Al Dhafer H, Fadl H., Abdeldayem M., El Torkey A. & El Gharbawy A. leg.; SW; J. Háva det.; KSMA; • 2 ex; Baha Province, Al Baha, Shabreqah, Baha Station, 20°08.034'N, 41°23.597'E; Alt. 2014 m; 24.iv.2013; Al Ansi A. & Al Harbi M. leg.; BS; J. Háva det.; KSMA • 1 ex; ibidem; Town Center, King Saud Road, 20°00.284'N, 41°27.912'E; Alt 2132 m; 25.iv.2013;Al Ansi A. & Al Harbi M. leg.; BS; J. Háva det.; KSMA • 1 ex; Baha Province, Belgershi, Elqamh park, 19°48.407'N, 41°12.738'E; Alt 1931 m; 17.v.2010; Dr. M R. Sharaf leg.; J. Háva det.; KSMA • 1 ex; Baha Province, Mukhwah, Shada Al Ala, 19°51.762'N, 41°18.089'E; Alt 1225 m; 16.ii.2014; Abdeldayem M. & Rasool I. leg.; HP; J. Háva det.; JHAC • 1 ex; ibidem; 19°50.575'N, 41°18.691'E; Alt 1666 m; 4.v.2015; Al Dhafer H., Fadl H., Abdeldayem M., El Torkey A.& El Gharbawy A. leg.; KSMA; • 1 ex; ibidem; Thee Ain Village, 19°55.727'N, 41°26.462'E; Alt 754 m; 15.v.2011; H. Al Fadly, A El Torkey, M. Sharaf & H. Setyaningrum leg.; SW; J. Háva det.; JHAC • 1 ex; Riyadh Province, Diriah, Al Ammariyah; 3.v.2011; Koko leg.; LT; J. Háva det.; KSMA • 1 ex; ibidem; Al Uyanah; 28.4.2010; Hazari T. leg.; SW; J. Háva det. KSMA • 1 ex; Riyadh Province, Diriah; 17.iii; SW (Dill); KSMA • 2 ex; ibidem; 24.iii; (Dill); KSMA

##### Remarks.

Háva and Herrmann (2009) initially identified *A.barbieri* as *A.heydeni* (Reitter, 1881) in Saudi Arabia. However, *A.heydeni* is not part of the Saudi Arabian fauna.

##### Distribution.

The species is found at altitudes ranging from 661 to 2717 m a.s.l. It is distributed across both lowland areas in the central region (Riyadh Province) and the mountainous regions in the southwest (Asir and Baha provinces) of Saudi Arabia (Fig. 4). Globally, it is known from Egypt, Israel, Jordan, and Palestine, and is new to Saudi Arabia.

#### ﻿ Attagenus beali

Taxon classificationAnimaliaColeopteraDermestidae

Zhantiev, 2005

E2056CFE-CBB0-5698-9B14-2DC76D59DD5E

Fig. 2C

##### Previous records.

Al Hufuf (Zhantiev 2005) and Udhailiyah Camp (Háva 2011) in the Eastern Province.

##### Material examined.

Saudi Arabia • 9 ex; Riyadh Province, Al Aflag, Al Naifiah, Farshet Sheaal, 22.40962°N, 46.59249°E; Alt. 601 m; 12.iv.2015; Abdeldayem M. et.al. leg.; LT; J. Háva det.; KSMA • 1 ex; ibidem; 22.40268°N, 46.59245°E; Alt. 588 m; 13.iv.2015; Abdeldayem M. et.al. leg.; PT; J. Háva det.; KSMA • 1 ex; ibidem; 22.41558°N, 46.558812°E; Alt. 599 m; 13.iv.2015; Abdeldayem M. et.al. leg.; PT; J. Háva det.; KSMA • 1 ex; ibidem; 22.43200°N, 46.58500°E; Alt. 597 m; 13.iv.2015; Al Dhafer H., Abdeldayem M., El Torkey A. El Gharbawy A., & Soliman A. leg.; PT; KSMA • 4 ex; Riyadh Province, Az Zulfi, Rawdat Al Sabalh, 26°21.792'N, 44°58.801'E; Alt. 666 m; 19.v.2015; M.S. Abdel-Dayem et al., leg.; LT; KSMA • 3 ex; ibidem; J. Háva det.; JHAC • 1 ex; ibidem; 26°22.429'N 44°58.241'E; Alt. 670 m; 20.v.2015; M.S. Abdel-Dayem et al., leg.; PT; J. Háva det.; KSMA • 2 ex; ibidem; 26°21.228'N, 44°58.999'E; Alt. 678 m; 26.viii.2015; Al Dhafer H., Fadl H., Abdeldayem M., El Gharbawy A., El Torkey A. & Soliman A. leg.; LT; J. Háva det.; KSMA • 3 ex; ibidem; 25.x.2015; Al Dhafer H., Fadl H., Abdeldayem M., El Gharbawy A., El Torkey A. & Soliman A. leg.; LT; J. Háva det.; KSMA • 1 ♀; Riyadh Province, Wadi ad-Dawasir, Uruq Bani Ma´arid, Site A, W. Merakha, 19°44'42"N, 45°11'52"E; Alt. 825 m; 6.iv.2021; Al Dhafer H., Soliman A. & Rassol I. leg.; LT; J. Háva det.; KSMA.

##### Remarks.

The species was first described from Saudi Arabia by Zhantiev in 2005, with subsequent records published by Háva (2011). This report provides additional locality records for the species.

##### Distribution.

This species is recorded at altitudes from 153 to 825 m a.s.l. It is found in the central region (Riyadh Province) and the eastern region (Eastern Province) of Saudi Arabia (Fig. 4). Globally, its distribution is confined to the Arabian Peninsula, with confirmed occurrences in Oman, Saudi Arabia, the United Arab Emirates, and Yemen.

#### ﻿ Attagenus chakouri

Taxon classificationAnimaliaColeopteraDermestidae

Pic, 1907

07059D69-1997-5BBC-94D9-22138D8C420F

Figs 2D, E

##### Previous records.

Saudi Arabia (Háva 2015b).

##### Material examined.

Saudi Arabia • 1 ♀; Riyadh Province, Ad Diriyah, 17.iii; (Dill); J. Háva det.; KSMA; • 2 ♀; ibidem; (Watercress); J. Háva det., KSMA; • 4 ex; ibidem; (Flowering Watercress); J. Háva det.; KSMA • 1 ♂; ibidem; 24.iii, (Dill); J. Háva det.; KSMA • 2 ♂; ibidem; (Flowering Watercress); J. Háva det.; KSMA • 1 ♂; 29.iii; (Flowering Watercress); J. Háva det.; KSMA • ♀ ex; 1941; (Casuarina); KSMA • 7 ex; without label; KSMA. • 7 ex; Riyadh Province, Huraymila, Wadi Hurayamala; 770 m; 10.iii.1988; C. Mills, Y. Aldryhim & A. Al-Dawood lgt.; J. Háva det.; FSCA.

##### Distribution.

In Riyadh Province in the central arid region of Saudi Arabia (Fig. 5), it is observed between 661 and 770 m a.s.l. Globally, it is known from Egypt, India (Uttar Pradesh), Iran, Iraq, Pakistan and Saudi Arabia.

#### ﻿ Attagenus cyphonoides

Taxon classificationAnimaliaColeopteraDermestidae

Reitter, 1881

94EB001A-E098-55C9-ABDF-749A55BFC817

Fig. 2F

##### Previous records.

Saudi Arabia (Hagstrum and Subramanyam 2009); Saudi Arabia (Háva 2015b).

##### Material examined.

Saudi Arabia • 1 ♀; Riyadh Province, Shaqra; 17.ii.1978; (Mill waste); J. Háva det.; KSMA.

##### Distribution.

Confined to the arid region of central Saudi Arabia, particularly in Riyadh Province (Fig. 5) at an elevation of 709 m a.s.l. The species is known from Europe, Egypt, Morocco, Nigeria, Senegal, South Sudan, Sudan, Tunisia, Afghanistan, China, India, Iran, Iraq, Israel, Kazakhstan, Mongolia, Pakistan, Palestine, Russia, Saudi Arabia, Tajikistan, Turkmenistan, Uzbekistan, Canada, Mexico, the United States, and Australia: Queensland (introduced).

#### ﻿ Attagenus fasciatus

Taxon classificationAnimaliaColeopteraDermestidae

(Thunberg, 1795)

A3651327-E64A-507D-B533-63ACFFE65B79

Fig. 2G, H

##### Previous records.

Shalaby (1961) reported the species in Jeddah as *A.gloriosus*, while Mroczkowski (1979) identified specimens from Taif and Riyadh as *A.cinnamomeus*. Additionally, Mroczkowski (2002) recorded specimens from Jeddah as *A.fasciatus*.

##### Material examined.

99 specimens listed in Suppl. material 1.

##### Remarks.

Shalaby (1961) documented the occurrence of *A.gloriosus* in Jeddah, while Mroczkowski (1979) also recorded *A.cinnamomeus* from Riyadh and Taif. Both *A.cinnamomeus* and *A.gloriosus* are now recognized as synonyms of *A.fasciatus*.

##### Distribution.

The species is locally widespread across different elevation ranges (26–1652 m a.s.l.). The distribution spans different regions of Saudi Arabia, including the central regions (Qassim and Riyadh provinces), the eastern region (Eastern Province), the western regions (ranging from Jazan in the south to Tabouk in the north), and the northern region (Jouf Province) of Saudi Arabia (Fig. 5). *Attagenusfasciatus* is a cosmopolitan species.

#### ﻿ Attagenus jakli

Taxon classificationAnimaliaColeopteraDermestidae

Háva, 2021

0CF9AE49-EC21-5D47-B590-7914EC6B2260

Fig. 2I

##### Material examined.

Saudi Arabia • 1 ♀; Baha Province, Al-Mandaq, W.Tourbah; 3.vi.2012; HP; Al Ansi A., Kondratieff B., Al Dhafer H. leg.; J. Háva det.; KSMA • 1 ex; Baha Province, Al-Mukhwah, Shada Al Ala, 19°51.762'N, 41°18.089'E; 1225 m; 2.iii.2015; LT; El Torkey A., El Gharbawy A., Mostafa A., Al Ansi A. & Rasool I. leg.; J. Háva det.; KSMA 1 ex; ibidem; JHAC • 2 ex; ibidem; 21.iv.2014; LT; Al Dhafer H., Fadl H., Abd el Dayem, M., El Torkey A. & El Gharbawy A. leg.; KSMA • 1 ex; ibidem; 19°51.066'N, 41°18.037'E; 1325 m, 21.iv.2014; LT; Al Dhafer H., Fadl H., Abd el Dayem, M., El Torkey A. & El Gharbawy A. leg., KSMA • 1 ex; ibidem; 19°50.329'N, 41°18.604'E; 1563 m; 21.iv.2014; LT; 21.iv.2014; LT; Al Dhafer H., Fadl H., Abd el Dayem, M., El Torkey A. & El Gharbawy A. leg., KSMA • 1 ♀; ibidem; 5.v.2015; LT; Al Dhafer H., Fadl H., Abd el Dayem, M., El Torkey A. & El Gharbawy A. leg.; J. Háva det.; JHAC.

##### Distribution.

**Figure 3.**
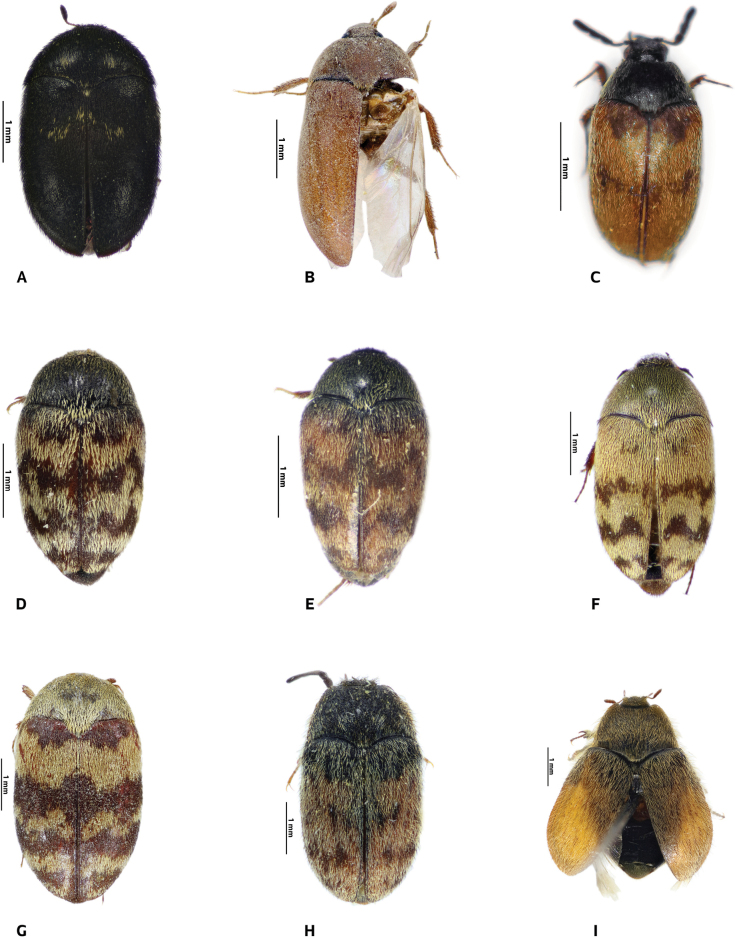
Dorsal habitus **A–G***Attagenus* species **A***A.kadleci* Háva, 2012 female **B***A.lobatus* Rosenhauer, 1856 female **C***A.logunovi* Háva, 2015 **D***A.posticalis* Fairmaire, 1878 **E***A. A.scalaris* (Pic, 1894) **F***A.vanharteni* Háva, 2009 **G***A.yemensis* Háva & Herrmann, 2014 **H***Telopestessellatus* Reitter, 1887 male **I***T.tessellatus* Reitter, 1887 female.

The species is restricted to the mountainous regions (1225–1600 m a.s.l.) of southwestern Saudi Arabia, particularly Baha Province (Fig. 3). It is endemic to the Arabian Peninsula, described from Oman, and is here reported for the first time in Saudi Arabia.

**Figure 4.**
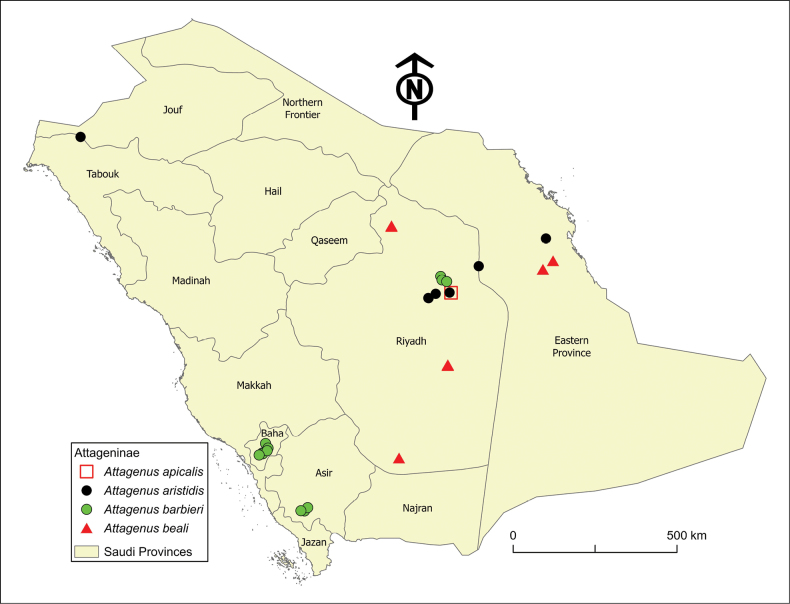
Distribution map of the *Attagenus* species in Saudi Arabia: *A.apicalis*, *A.aristidis*, *A.barbieri*, and *A.beali*.

#### ﻿ Attagenus kadleci

Taxon classificationAnimaliaColeopteraDermestidae

Háva, 2012

C8F5BD82-D377-510A-8A06-55CE2990D348

Fig. 3A

##### Material examined.

Saudi Arabia • 1 ♀; Asir Province, Abha, Raydah, 18°11.766'N 42°24.315'E; 2285 m; 5.ix.2015; SW; Al Dhafer H., Fadl H., Abdel-Dayem M., El Gharbawy A., El Torkey A. & Soliman A.; J. Háva det.; JHAC.

##### Distribution.

**Figure 5.**
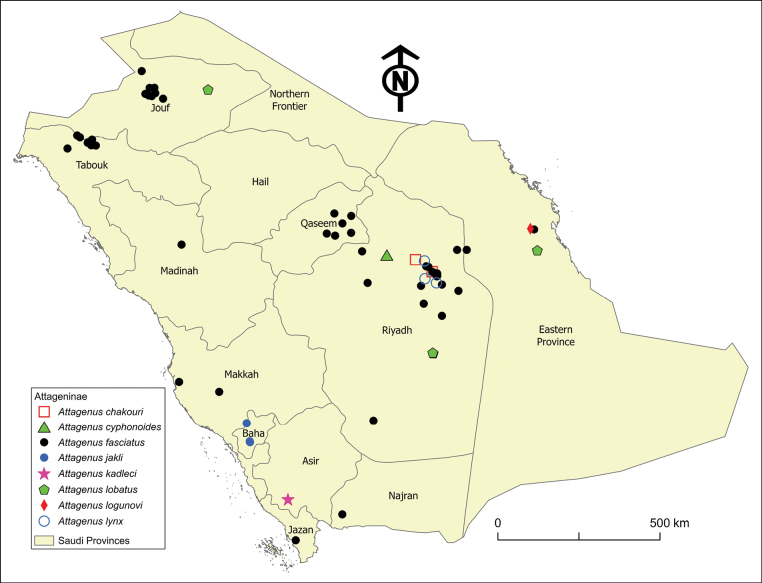
Distribution map of *Attagenus* species in Saudi Arabia: *A.chakouri*, *A.cyphonoides*, *A.fasciatus*, *A.jakli*, *A.kadleci*, *A.lobatus*, *A.logunovi*, and *A.lynx*.

It may be a montane species found at an elevation of 2285 m a.s.l. in the southwestern region of Saudi Arabia, especially Asir Province (Fig. 5). *Attagenuskadleci* is endemic to the Arabian Peninsula, previously described from Yemen, and is here newly recorded in Saudi Arabia.

#### ﻿ Attagenus lobatus

Taxon classificationAnimaliaColeopteraDermestidae

Rosenhauer, 1856

6304165A-2AB6-5F79-9E03-5967A1C9B13F

Fig. 3B

##### Previous records.

Hofuf in the Eastern Province, Hedjaz (Mroczkowski 1979), and Saudi Arabia (Hagstrum and Subramanyam 2009).

##### Material examined.

Saudi Arabia • 1 ex; Jouf Province, Sakaka, Salem Al Salem Farm, 30°00'40.2"N, 40°05'58.7"E; 21.vi.2022; SW (Parsley); A. Al Ansi et al. Leg.; M.S. Abdel-Dayem det.; KSMA • 1 ex; Riyadh Province, Al-Aflag, Al Naifiyah, Farshet Sheal; 22.4279°N, 46.5747°E; 612 m; 13.iv.2015; PT (Acacia); Al Dhafer H., Fadl H., Abdel-Dayem M., El Gharbawy A., El Torkey A. & Soliman A. Leg.; M.S. Abdel-Dayem det.; KSMA • ♀; J. Háva det.; KSMA.

##### Remarks.

The records from Rawdhat Khorim (Al Dhafer et al. 2016; Abdel-Dayem et al. 2017) and Wadi Hanifa (Abdel-Dayem et al. 2023) should be attributed to *A.vanharteni*.

##### Distribution.

The species is locally distributed within Saudi Arabia in the central region (Riyadh Province), the eastern region (Eastern Province), and the northern region (Jouf Province) (Fig. 5). It inhabits elevations from 153 to 605 m a.s.l. Globally, its distribution spans a wide range of habitats, from deserts to temperate zones. The species is known from Europe (Bulgaria, Czech Republic, France, Greece, Italy, Romania, Sardinia, Spain, Turkey, Ukraine), Africa (Algeria, Egypt, Morocco, Sudan, Tunisia), Asia (Afghanistan, China, India, Iran, Iraq, Kazakhstan, Kyrgyzstan, Mongolia, Pakistan, Russia, Saudi Arabia, Syria, Tajikistan, Turkmenistan, United Arab Emirates, and Uzbekistan), and North America (U.S.A.).

#### ﻿ Attagenus logunovi

Taxon classificationAnimaliaColeopteraDermestidae

Háva, 2015

64A0B329-B6D4-555B-81ED-DB4B1516FFF5

Fig. 3C

##### Previous records.

Eastern Province, Ain Dar (Háva 2015a).

##### Material examined.

Saudi Arabia • 7 ♀, 2 ♂; Eastern Province, Ain Dar; 25.991°N, 49.389°E; Alt. 146 m; 16.iv.1982; Pitcher D.A. leg.; J. Háva det.; MMUE • 7 ♀, 2 ♂; ibidem; 16.iv.1982; Pitcher D.A. leg.; J. Háva det.; JHAC.

##### Distribution.

The species is endemic to Saudi Arabia, found at an elevation of 146 m a.s.l. Its existence is restricted to the eastern region, specifically the Eastern Province (Fig. 5).

#### ﻿ Attagenus lynx

Taxon classificationAnimaliaColeopteraDermestidae

(Mulsant & Rey, 1868)

95417F3D-9491-5FD2-B357-772C14518377

##### Previous records.

Wadi Mizbil, Wadi Salbukh, Wadi Shaib Luha (Mroczkowski 1979) in Riyadh Province.

##### Remarks.

The material mentioned by Mroczkowski (1979) needs to be examined, as it probably belongs to a different species. Specimens from Ibex Reserve, initially noted by Al Dhafer et al. (2012), were subsequently identified as *A.scalaris*. Similarly, specimens from Wadi Hanifa, identified by Abdel-Dayem et al. (2023), were later determined to belong to *A.vanharteni* and *A.posticalis*.

##### Distribution.

The species is found in the arid areas within the central region (Riyadh Province) of Saudi Arabia (Fig. 5) between 560 and 680 m a.s.l. It is globally known from Armenia, Azerbaijan, Finland (introduced), Egypt, Iran, Israel, Kazakhstan, Mongolia, Pakistan, Palestine, Poland (introduced), Qatar, Russia, Saudi Arabia, Syria, Tadzhikistan, Turkey, Turkmenistan, and Uzbekistan.

#### ﻿ Attagenus pellio

Taxon classificationAnimaliaColeopteraDermestidae

(Linnaeus, 1758)

DCD8A50B-808A-51AE-9730-B097B69E5EC1

##### Previous records.

Saudi Arabia (Hagstrum and Subramanyam 2009)

##### Remarks.

Hagstrum and Subramanyam (2009) referenced the species without specifying its locality.

#### ﻿ Attagenus posticalis

Taxon classificationAnimaliaColeopteraDermestidae

Fairmaire, 1879

452FE661-8FE2-50C3-96CF-FD4BAA7A2C3C

Fig. 3D

##### Previous records.

The species has been reported from the Eastern Province, Al Uyun, Al Hasa (Háva 2011); Rhawdet Khorim (Al Dhafer et al. 2016; Abdel-Dayem et al. 2017); and the Farasan Archipelago, Farasan and Sajid islands (Abdel-Dayem et al. 2020). Although Mroczkowski (2002) reported Wattayah (23°36'N, 58°30'E) as a locality for this species in Saudi Arabia, this locality is situated in Oman, not Saudi Arabia.

##### Material examined.

242 specimens listed in Suppl. material 1.

##### Distribution.

**Figure 6.**
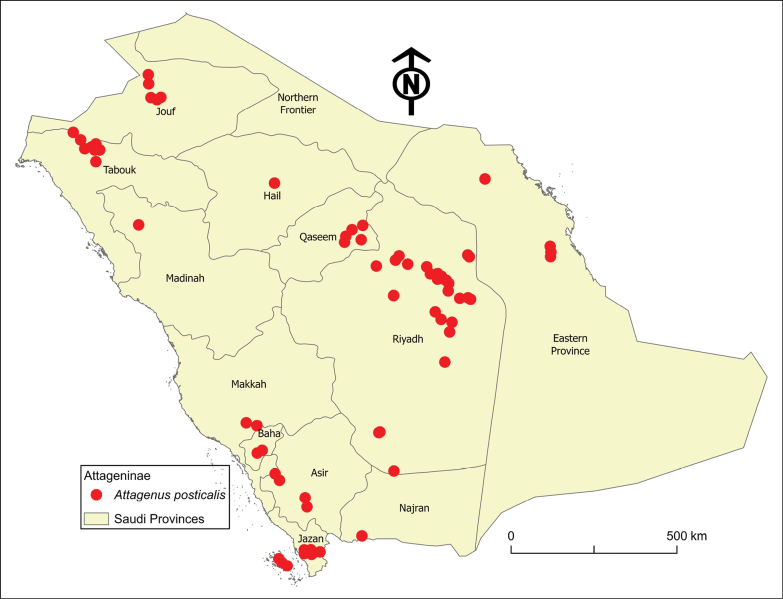
Distribution map of *Attagenusposticalis* in Saudi Arabia.

This species is the most prevalent and widely distributed member of the Attageninae in Saudi Arabia, found in almost all provinces (Fig. 6), with an elevation range of 6–1990 m a.s.l. Globally, its distribution spans the Afrotropical region, including Eritrea, Ethiopia, and Senegal. It extends northward into the Sahel region, encompassing Mauretania, Niger, and Sudan; as well as North Africa, including Algeria, Egypt, Morocco, and Tunisia; and reaches into southern Europe (Spain). To the east, it is found in the Levant region, which includes Jordan, Palestine, and Syria, as well as across the Arabian Peninsula, encompassing Kuwait, Oman, Qatar, Saudi Arabia, the United Arab Emirates, and Yemen.

#### ﻿ Attagenus scalaris

Taxon classificationAnimaliaColeopteraDermestidae

(Pic, 1894)

764F7D25-766F-563B-A75D-DF9AFFC97DEA

Fig. 3E

##### Previous records.

Riyadh (Alsuhaibani 1996) and Ibex Reserve (Al Dhafer et al. 2012 as *A.lynx*) in Riyadh Province.

##### Material examined.

Saudi Arabia • 1 ex; Baha Province, Al-Mukhwah, Shada Al Ala, 19°51.066'N, 41°18.037'E; Alt. 1325 m; 26.i.2015; PT; Al Dhafer H., Fadl H., Abd el Dayem, M., El Torkey A. & El Gharbawy A. leg.; J. Háva det.; KSMA • 1 ex; ibidem; 8.xii.2014; PT; Al Dhafer H., Fadl H., Abd el Dayem, M., El Torkey A. & El Gharbawy A. leg.; J. Háva det.; KSMA • 1 ex; ibidem; 19°50.710'N, 41°18.267'E; Alt. 1474 m; 3.vi.2014; LT; Al Dhafer H., Fadl H., Abd el Dayem, M., El Torkey A. & El Gharbawy A. leg.; J. Háva det.; KSMA • 1 ex; Jouf Province, Tabarjal, Basieta, NADEC, F138, 29°52.29'N, 38°18.403'E; Alt. 635 m; 24.v.2018, SW (Olive); Hathal et al. leg.; J. Háva det.; JHAC • 1 ex; Riyadh Province, Diriyah, Al Uyaynah, Wadi Hanifa, WHN02, 24.9119°N, 46.187792°E; Alt. 806.755 m; 29.iv.2015; PT; Abdel-Dayem M. et al. leg.; J. Háva det.; KSMA • 1 ex; Riyadh Province, Hutet Beni Tamem, Ibex Reserve National Park, W. 180 km S Riyadh Region; 20.x.2007, Al Doryhem Y., Aldhafer H., Almotairy M. & Algharbawy A. leg.; *Attagenuslynx*; J. Háva det.; KSMA • 1 ex; without data; M.S. Abdel-Dayem det.; KSMA.

##### Distribution.

**Figure 7.**
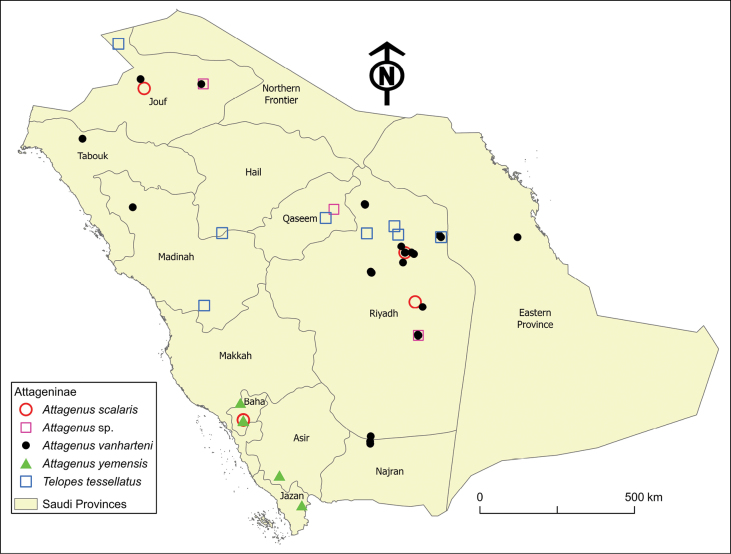
Distribution map of Attageninae species in Saudi Arabia: *Attagenusscalaris*, *A.* sp., *A.vanharteni*, *A.yemensis*, and *Teleopestessellatus*.

The species is found at elevations ranging from 635 to 1474 m a.s.l. across the central region (Riyadh Province), southwestern region (Baha Province), and northern region (Jouf) (Fig. 7). Globally, it is known from Egypt, Libya, Israel, Palestine, and Saudi Arabia.

#### ﻿ Attagenus smirnovi

Taxon classificationAnimaliaColeopteraDermestidae

Zhantiev, 1973

543F8FE0-087A-5EC4-878B-CB2FD89B357D

##### Previous records.

Saudi Arabia (Hagstrum and Subramanyam 2009)

##### Remarks.

Although Hagstrum and Subramanyam (2009) mentioned the species from Saudi Arabia, they did not provide specific details about its locality; from the Arabian Peninsula the species is known from Oman and Yemen

#### ﻿ Attagenus vanharteni

Taxon classificationAnimaliaColeopteraDermestidae

Háva, 2009

46576BE0-ED8D-5DF3-9F4C-A3C3966DBC36

Fig. 3F

##### Previous records.

The species was previously reported from Hofuf (Mroczkowski 1979 and Rhawdet Khorim (Al Dhafer et al. 2016; Abdel-Dayem et al. 2017) under the name *A.fasciolatus*.

##### Material examined.

126 specimens listed in Suppl. material 1.

##### Remarks.

Mroczkowski (1979) and Al Dhafer et al. (2016) previously reported the species in Saudi Arabia as *A.fasciolatus*. However, this species is excluded from the fauna of Saudi Arabia as the specimens reported actually belong to *A.vanharteni* Háva, 2009.

##### Distribution.

The species is found at elevations between 153 and 980 m a.s.l. It is recorded across different regions of Saudi Arabia, including the central region (Riyadh Province), the eastern region (Eastern Province), the northern regions (Jouf, Madinah, and Tabouk provinces) and the southern region (Najran Province) (Fig. 7). Globally, it is endemic to the Arabian Peninsula, with known occurrences in Oman, Qatar, the United Arab Emirates, and Yemen; and here it is newly recorded in Saudi Arabia.

#### ﻿ Attagenus yemensis

Taxon classificationAnimaliaColeopteraDermestidae

Háva & Herrmann, 2014

2EC91F70-2D8B-5F19-B22D-484DA32FA9C6

Fig. 3G

##### Material examined.

Saudi Arabia • 4 ex; Asir, Abha, Raydah, 18°11.618'N, 42°23.420'E; Alt. 1772 m; 8.vi.2014; LT 8; Al Dhafer H., Fadl H., Abdeldayem M., El Torkey A & El Gharbawy A. leg.; J. Háva det.; KSMA • 3 ex; Bahah Province, Al-Mukhwah, Shada Al Ala, 19°51.762'N, 41°18.089'E; Alt. 1225 m; 5.v.2015; LT 6; Al Dhafer H., Fadl H., Abdeldayem M., El Torkey A. & El Gharbawy A. leg.; J. Háva det.; KSMA • 1 ex; ibidem; 3.vi.2014; LT 4; J. Háva det.; JHAC • 1 ex; ibidem; 2.ix.2015; LT 2; J. Háva det.; KSMA • 4 ex; ibidem; 27.vii.2015; J. Háva det.; KSMA • 3 ex; Baha Province, Al-Mandaq, W.Tourbah; 3.vi.2012; HP; Al Ansi A., Kondratieff B. & Al Dhafer H. leg.; J. Háva det.; KSMA • 1 ex; ibidem; J. Háva det.; JHAC • 1 ex; Jizan Province, Al-Idabi, Aiban, W. Gowra, 17°17.569'N, 43°04.211'E; Alt. 451 m; 11.xi.2012; BS; H.H. Fadl leg.; J. Háva det.; KSMA.

##### Distribution.

This species is confined to the southwestern regions of Saudi Arabia, specifically Asir, Baha, and Jazan provinces (Fig. 7), and is found at altitudes ranging from 451 to 1897 m a.s.l. It is endemic to the Arabian Peninsula, described from Yemen, and here, newly recorded in Saudi Arabia.

#### ﻿ Attagenus

Taxon classificationAnimaliaColeopteraDermestidae

sp.

9A65081E-BDB5-5537-BBE0-185AA1ECC0F0

##### Material examined.

Saudi Arabia • 1 ♀; Jouf Province, Sakaka, Saleem Al Salim F., 30°00.407'N, 40°05.587'E; 21.vi.2022; SW (Parsely); A., Al Ansi et al. leg.; J. Háva det.; JHAC • 1 ♀; Qassim Province, Buraydah; 26°12.954'N, 44°02.483'E; Alt. 633 m; 17.ix.2011; SW; H. Setyaningrum & A. Al Ansi leg.; J. Háva det.; KSMA • 1 ♀; Riyadh Province, Al Aflag, Al Naifiyah, Farshet Sheaal, 22.4062°N, 46.59249°E; Alt. 601 m; 13.iv.2015; PT (*Leptadeniapyrotechnica*), Al Dhafer H., Abdeldayem M, El Torkey A., El Gharbawy A., Soliman A. leg.; J. Háva det.; KSMA.

##### Remarks.

These female specimens likely represent a new species, closely resembling *A.geisthardti* Herrmann & Háva, 2017. However, the species is not described in this study due to the unavailability of a male specimen, which is necessary for a comprehensive taxonomic description.

##### Distribution.

The species is found at elevations ranging from 601 to 633 m a.s.l. and is distributed in the central region (Qassim and Riyadh provinces) and the northern region (Jouf Province) of Saudi Arabia (Fig. 7).

###### ﻿Genus *Telopes* Redtenbacher in Russiger 1843

#### ﻿ Telopes tessellatus

Taxon classificationAnimaliaColeopteraDermestidae

Reitter, 1887

CBA44421-EC7A-58C7-B2CC-9D04AA4BF833

Fig. 3H, I

##### Previous records.

Rhodet Khorim in Riyadh (Abdel-Dayem et al. 2017 1979 as *A.reitteri*).

##### Material examined.

Saudi Arabia • 1 ♀; Hail Province, Al Hayit, 15 km S of Fanudah, 25°30'14.7"N, 40°39'48.6"E; 7.iii.2021, M. Šárovec lgt.; J. Háva det.; JHAC • 1 ♂; Jouf Province, Al-Qurrayat, Rashid Manateh Farm, 31°13'24.3"N, 37°31'45.1"E; 8.iii.2022; SW (Onion); A. Al Ansi et al. leg.; *Attagenusreitteri*; J. Háva det.; KSMA • 1 ex; Madinah Province, Madinah, Hazem, Awad Farm, 23°18'15.3"N, 40°07'17.6"E; Alt. 1026 m; 7.ii.2022; Sw (Tomato); *Attagenusreitteri*; J. Háva det.; KSMA • 1 ♂; Qassim Province, Al-Badai, Abdullah Ashimbani Farm, 25°57'25"N, 43°47'31"E; Alt. 700 m; 15.ii.2022; SW (Barley); H. Chebbi & H. Abbad leg.; *Attagenusreitteri*; J. Háva det.; KSMA • 1 ♂; Riyadh Province, Al-Majmaah, Tumayr, 25°42'36"N, 45°52'11"E; 1.ii.2010; MT; Al Dhafer H. & Al Husein F. leg.; *Attagenusreitteri*; J. Háva det.; KSMA • 2 ♂; ibidem; 8.ii.2010; MT; Al Dhafer H. & Al Husein F. leg.; *Attagenusreitteri*; J. Háva det.; KSMA • 2 ex; ibidem; JHAC • 1 ♀; ibidem; 15.ii.2010; *Attagenusreitteri*; J. Háva det.; KSMA • 1 ♂; ibidem; 26.ii.2010; *Attagenusreitteri*; J. Háva det.; KSMA • 1 ♂; Riyadh Province, Ramah, Rhodet Khorim, 25°22.986'N, 47°16.712'E; Alt. 559 m; 9.ii.2013, SW (*Rhazyasteicta*); *Attagenusreitteri* Mroczkowski, 1968; J. Háva det.; KSMA • 1 ♂; Riyadh Province, Shaqra, 20 km NW Ushaiqur, 25°29'47.9"N, 45°02'14"E; 5–6.iii.2023, M. Šárovec lgt.; J. Háva det.; JHAC • 1 ♀; Riyadh Province, Thadiq, cca 90 km NNW of Riyadh, 25°27'25.4"N, 45°59'5.6"E; 5.iii.2023; M. Krejčíř lgt.; J. Háva det.; JHAC.

##### Remarks.

The species was previously misidentified as *Attagenusreitteri* Mroczkowski, 1968 by Abdel-Dayem et al. (2017). However, that taxon is now excluded from the fauna of Saudi Arabia.

##### Distribution.

This species, new to Saudi Arabia, is found in the central region (Hail, Qassim, and Riyadh provinces), the western region (Madinah Province), and the northern region (Jouf Province) (Fig. 7). Its elevation ranges from 518 to 1136 m a.s.l. Globally, it is distributed across the Mediterranean Basin, including Egypt, Libya, Israel, Palestine, Syria, and Turkey.

## ﻿Discussion

This study explores the subfamily Attageninae (Coleoptera: Dermestidae) in Saudi Arabia, focusing on expanding species records, exhibiting distributional patterns, and revising historical records. Twenty species from two genera (*Attagenus* and *Telopes*) were documented, and six were reported for the first time in the country. This Attageninae species richness represents a significant portion (26.7%) of the known Palaearctic fauna, which comprises ~ 75 species across five genera (Háva 2015b, 2024). The endemic Attageninae species comprises ~ 1% (one species) and 18.5% (5 species) of the known Saudi and Arabian faunas, respectively. The findings of this study contribute to our understanding of the Attageninae fauna in Saudi Arabia and the Arabian Peninsula. The Arabian Peninsula in general and Saudi Arabia in particular is home to a remarkable biodiversity (Mallon 2011), which supports the importance of further the necessity of further exploration and taxonomic studies. The new records of *A.barbieri*, *A.jakli*, *A.kadleci*, *A.vanharteni*, *A.yemensis*, and *Telopestessellatus* mark a notable increase in the known Attageninae species by 43% in Saudi Arabia and 7% in the Arabian Peninsula. The inclusion of these species highlights Saudi Arabia’s biogeographic importance as a bridge connecting the Afrotropical and Palaearctic regions, as previously documented by El-Hawagry et al. (2016) and Abdel-Dayem et al. (2017, 2020).

Based on morphological examinations, five species (*A.dichrous*, *A.fasciolatus*, *A.heydeni*, *Telopesobtusus*, and *Telopesreitteri*) previously reported in the Attageninae fauna of Saudi Arabia were excluded due to the incorrect identification (Mroczkowski 1979; Háva and Herrmann 2009; Al Dhafer et al. 2016; Abdel-Dayem et al. 2017). This emphasizes the critical need for ongoing taxonomic research. Despite prior reports of *A.apicalis*, *A.atripennis*, *A.lynx*, *A.pellio*, and *A.smirnovi* (Mroczkowski 1979; Hagstrum and Subramanyam 2009), no specimens have been collected in subsequent surveys. Thus, the occurrence status of these species in Saudi Arabia remains unknown, requiring further research to determine their rarity or local extinction.

Attageninae species exhibit distinct altitudinal distributions in Saudi Arabia, reflecting their ecological adaptations to varying environmental conditions. The exclusive presence of *A.apicalis* and *A.logunovi* in low-altitude regions (≤ 600 m) highlights their adaptation to arid and semi-arid lowland environments. Such habitat specificity is aligned with global observations of lowland-adapted species, where factors like high temperatures, aridity, and specific vegetation types create niche environments (Konstantinov et al. 2009; Sømme 2012). On the other hand, *A.kadleci* may certainly be restricted to high-altitude ecosystems, reflecting a distinct preference for montane ecosystems. This altitudinal limitation emphasizes its adaptation to cooler, high-altitude environments, aligning with similar observations documented in Yemen (Háva 2012). The mid-altitude range emerges as a zone of highest species richness, hosting 70% (14 species) of Attageninae. This richness likely results from the transitional nature of mid-altitudes, which bridge the distinct ecological characteristics of lowlands and highlands. Mid-altitudes offer more diverse environmental gradients (e.g., temperature, moisture, and vegetation) that create multiple niches conducive to species coexistence. These results align with ecological theories predicting a mid-elevation peak in biodiversity due to overlapping environmental conditions (McCain and Grytnes 2010). Generalist species (e.g., *A.fasciatus*, *A.posticalis*, and *A.yemensis*) exhibit considerable ecological plasticity, thriving across lowland to highland habitats. This adaptability may be attributed to broader physiological tolerances, diverse feeding habits, or reproductive strategies that enable survival across varying conditions (Hodkinson 2005). These findings highlight the role of generalist traits in maintaining biodiversity in regions with dynamic environmental gradients (Carnicer et al. 2012). Climate change could significantly impact this altitudinal distribution of Attageninae species in Saudi Arabia, potentially causing range shifts or local extinctions (McCain and Garfinkel 2021), especially for altitude-restricted species like *A.kadleci*. Additionally, increasing anthropogenic activities, such as urbanization and land-use changes, may further fragment habitats and disrupt the distribution patterns of Attageninae species (Miličić et al. 2021), particularly in mid-altitude regions where species richness is highest.

From a conservation perspective, approximately 40% of Attageninae species in Saudi Arabia are conserved within the country’s protected areas. The Graf Raydah Nature Reserve in Asir Province provides a habitat for *A.barbieri*, *A.kadleci*, and *A.yemensis*, species associated with montane environments (Háva 2012, 2021; Háva and Herrmann 2014; Abdel-Dayem et al. 2018). The Shada Al Ala Nature Reserve in Baha Province supports a higher Attageninae species richness, including *A.barbieri*, *A.jakli*, *A.posticalis*, *A.scalaris*, and *A.yemensis*. This nature reserve is recognized as a biodiversity hotspot, supporting a diverse species assemblage (El-Hawagry et al. 2016; Abdel-Dayem et al. 2019). The presence of *A.scalaris* in the Ibex Reserve highlights the importance of preserving arid and semi-arid ecosystems to sustain such specialized fauna (Al Dhafer et al. 2012). Meanwhile, Rhawdet Khorim National Park sustains populations of *A.fasciatus*, *A.posticalis*, and *A.vanharteni*, demonstrating its role in conserving species with both endemic and broader distribution ranges (Al Dhafer et al. 2012; Abdel-Dayem et al. 2017). These findings underscore the effectiveness of protected areas in Saudi Arabia in conserving endemic and regionally significant Attageninae species and their habitats.

The Attageninae fauna of Saudi Arabia demonstrates notable similarities and distinctions compared to adjacent countries within the Arabian Peninsula. Studies indicate that Saudi Arabia hosts most of the Attageninae species documented in the region, accounting for approximately 74% of the known species (Háva 2021, 2024). Comparatively, Saudi Arabia shares notable similarities of its Attageninae with those of Yemen (40%), Oman and the United Arab Emirates (35% for each), Qatar (20%), and Kuwait (15%) (Háva 2024). This emphasizes Saudi Arabia’s potential for revealing unique species and, moreover, extending the known ranges of Attageninae.

## ﻿Conclusions

This study contributes to our understanding of the Attageninae fauna in Saudi Arabia by addressing taxonomic gaps, expanding the catalog of recorded species, and providing detailed distributional data. The results emphasize the critical need for systematic biodiversity research in the region. Future studies should focus on molecular phylogenetic approaches and explore the ecological roles of these species to gain a more comprehensive understanding of Attageninae diversity and its role in ecosystem functioning.

## Supplementary Material

XML Treatment for Attagenus apicalis

XML Treatment for Attagenus aristidis

XML Treatment for Attagenus atripennis

XML Treatment for Attagenus barbieri

XML Treatment for Attagenus beali

XML Treatment for Attagenus chakouri

XML Treatment for Attagenus cyphonoides

XML Treatment for Attagenus fasciatus

XML Treatment for Attagenus jakli

XML Treatment for Attagenus kadleci

XML Treatment for Attagenus lobatus

XML Treatment for Attagenus logunovi

XML Treatment for Attagenus lynx

XML Treatment for Attagenus pellio

XML Treatment for Attagenus posticalis

XML Treatment for Attagenus scalaris

XML Treatment for Attagenus smirnovi

XML Treatment for Attagenus vanharteni

XML Treatment for Attagenus yemensis

XML Treatment for Attagenus

XML Treatment for Telopes tessellatus

## ﻿Additional information

### Author ORCIDs

Jiří Háva https://orcid.org/0000-0001-8076-9538

Mahmoud S. Abdel-Dayem https://orcid.org/0000-0002-6276-1740

Hathal M. Aldhafer https://orcid.org/0000-0002-4911-2332

### Data availability

All of the data that support the findings of this study are available in the main text or Supplementary Information.

## Supplementary materials

Supplementary material 1 *Attagenusfasciatus* (Thunberg, 1795) Data type docx https://binary.pensoft.net/file/1360905This dataset is made available under the Open Database License (http://opendatacommons.org/licenses/odbl/1.0/). The Open Database License (ODbL) is a license agreement intended to allow users to freely share, modify, and use this Dataset while maintaining this same freedom for others, provided that the original source and author(s) are credited.Jiří Háva, Mahmoud S. Abdel-Dayem, Hathal M. Aldhafer
