# Supplementary material for: ﻿The subfamily Attageninae (Coleoptera, Dermestidae) from Saudi Arabia
Source: Zookeys. 2025 Jun 25;1243:107–30. doi: 10.3897/zookeys.1243.146325 (PMC12238960; doi:10.3897/zookeys.1243.146325)
Supplement: Supplementary material 1 — Attagenusfasciatus (Thunberg, 1795) [file zookeys-1243-107_article-146325__-s001.docx]

**Supplementary File**

***Attagenus fasciatus* (Thunberg, 1795)**

**Material examined.** SAUDI ARABIA • 1 ♂; Eastern Province, Buqayq, Abquiq; 3.xi.1983; No329; D.A. Pitcher leg.; J. Háva det.; JHAC • 1 ex; Jazan Province, Abu Arish, F89, 17°01.218'N, 42°37.643'E; Alt. 26 m; 11.v.2018; SW; H. Aldhafer et al. Leg.; J. Háva det.; KSMA • 1 ex; Jouf Province, Sakaka, Eqab Khalaf Alsalem Farm, 29.943357°N, 40.32668°E; Alt. 599 m; 21.v.2024; SW (Olive); A. Ishag & N. Elgoni leg.; M.S. Abdel-Dayem det.; KSMA • 1 ex; Jouf Province, Tabarjal, Abdul-Mohsin Al-Sarab Farm, 30°31'45.5"N, 38°11'21.4"E; 27.v.2022; PH (Peach); A, Al Ansi et al. Leg.; M.S. Abdel-Dayem det.; KSMA • 1 ex; ibidem; Basieta, JADCO_2 (F128), 30°48.791'N, 38°28.604'E; Alt. 625 m; 21.v.2018; VC (Barsem); H. Aldhafer et al. Leg.; M.S. Abdel-Dayem det.; KSMA • 1 ex; ibidem; JADCO (F125), 30°03.08'N, 38°33.266'E; Alt. 613 m; 23.v.2018; VC (Alfalfa); H. Aldhafer et al. Leg.; M.S. Abdel-Dayem det.; KSMA • 3 ex; ibidem; JADCO_6 (F126), 30°15.598'N, 38°14.616'E; Alt. 587 m; 23.v.2018, SW; H. Aldhafer et al. Leg.; M.S. Abdel-Dayem det.; KSMA • 5 ex; ibidem; JADCO_6 (F139), 30°02.951'N, 38°24.908'E; Alt. 607 m; 26.v.2018, SW (Sudanies Maiz); H. Aldhafer et al. Leg.; J. Háva det.; KSMA • 2 ex; ibidem; NADEC (F136), 29°52.453'N, 38°17.592'E; Alt. 631; 24.v.2018, SW; H. Aldhafer et al. Leg.; M.S. Abdel-Dayem det.; KSMA • 1 ex; ibidem; Watania, 29°53'51.8"N, 38°34'46.8"E; 21.vii.2022, PH (Peach); A. Al Ansi et al. Leg.; M.S. Abdel-Dayem det.; KSMA • 2 ex; ibidem; Mequa, Jana Agriculture Farm, 29°44'13.1"N, 38°48'11.8"E; 19.vi.2022, PH (Fig); A, Al Ansi et al. Leg.; J. Háva det.; KSMA • 1 ex; Madinah Province, Khaybar, Husain Fares Farm (F5), 25.53494°N, 39.3372°E; Alt. 752 m; 28.iii.2021, SW (Grass under date palm); H. Aldhafer et al. Leg.; J. Háva det.; KSMA • 1 ex; Najran Province, Hubuna, Al-Majma, Salih Al-Jaeim Farm, 17°45'40.6"N, 43°58'14.6"E; 16.ix.2021, SW, Medicago sativa, U. Abu El-Ghiet & B. Busalem leg.; J. Háva det.; JHAC • 1 ex; Qassim Province, Al-Rass; Hatan leg.; M.S. Abdel-Dayem det.; KSMA • 1 ex; Qassim Province, Al-Shamasiyah, Mohammed Farm, 26°21'27"N, 44°13'39"E; Alt. 599 m; 8.ix.2021; SW (Clover); H. Chebbi & H. Abbad leg.; J. Háva det.; KSMA • 1 ex; Qassim Province, Buraidah,Al Qaraa, Al Hijrisyyah Farm (F64), 26°25.697'N, 43°44.36'E; Alt. 599 m; 4.v.2018; SW; H. Aldhafer et al. Leg.; M.S. Abdel-Dayem det.; KSMA • 1 ex; Qassim Province, Mithnab, Husein Al Beheiri Farm (F50), 25°52.381'N, 44°13.353'E; Alt. 636 m; 2.v.2018; SW; H. Aldhafer et al. Leg.; M.S. Abdel-Dayem det.; KSMA • 1 ex; Qassim Province, Unayzah, Al Rawghani Farm (F42), 26°08.742'N, 43°58.396'E; Alt. 669 m; 2.v.2018; VC; H. Aldhafer et al. Leg.; J. Háva det.; KSMA • 1 ex; Qassim Province, Unayzah, Al Wadi, Mubarak bin M. Al Qadeem Farm (F40), 25°47.429'N, 43°45.647'E; Alt. 711 m; 01.v.2018; SW; H. Aldhafer el al. Leg.; J. Háva det.; KSMA • 1 ex; Riyadh Province, Ad Duwadimi, Sajir, Al Mushawah Farm (F36), 25°20.36'N, 44°32.401'E; Alt. 700 m; 29.iv.2018; SW; H. Aldhafer et al. Leg.; J. Háva det.; KSMA • 3 ex; ibidem; VC; H. Aldhafer et al. Leg.; M.S. Abdel-Dayem det.; KSMA • 3 ex; Riyadh Province, Ad Duwadimi, Al Hafira; 15.x.2003; Abdullah Al Yaqub leg.; M.S. Abdel-Dayem det.; KSMA • 1 ex; Riyadh Province, Al Muzahimiyah, Khararah, 24.35°N, 46.24°E; 15.ix.2013; LT; Khan J., Zahour U., Khan I. & Kamran A.; J. Háva det.; KSMA • 1 ex; Riyadh Province, Diriyah, Al Uyaynah; 28.iv.2010; SW; Al-Dossary M. Leg.; J. Háva det.; KSMA • 1 ex; ibidem; 12.v.2010; SW; Al-Hashel A. Leg.; J. Háva det.; KSMA • 1 ex; ibidem; 3.x.1991; SW (Flax); M.S. Abdel-Dayem det.; KSMA • 1 ex; ibidem; Jobilah, 45 km W Riyadh, Salam Farm; 8.ix.2007; LT; Otybi Mohomumad leg.; J. Háva det.; KSMA • 1 ex; Riyadh Province, Diriyah; 24.ix.1991; SW (Flax); M.S. Abdel-Dayem det.; KSMA • 1 ex; ibidem; 12.x.2011; SW (Flax); A. Alomar leg.; M.S. Abdel-Dayem det.; KSMA • 1 ex; ibidem; 16.x.2000; M. Alkhurigi leg.; *Attagenus gloriosus*; M.S. Abdel-Dayem det.; KSMA • 1 ex; Riyadh Province, Hair; 28.i.1980; SW (Alfalfa); M.S. Abdel-Dayem det.; KSMA • 1 ex; Riyadh Province, Howtat Bani Tamim, 23°49'35"N, 46°18'58"E; 15.iv.2010; SW (Alfalfa); Abdulah Al-Othman leg.; M.S. Abdel-Dayem det.; KSMA • 1 ex; ibidem; Saoud Mohammed Al Taleb Farm, 23°28'37"N, 46°50'31"E; Alt. 563 m; 22.ix.2021; SW (Alfalfa); H. Chebbi & H. Abbad leg.; J. Háva det.; KSMA • 1 ex; Riyadh Province, Kharj; 2.v.2007; M.S. Abdel-Dayem det.; KSMA • 1 ex; Riyadh Province, Ramah, Rhodet Khorim; Alt. 559 m; 1.iv.2012; LT (A); *Attagenus smirnovi*; J. Háva det.; KSMA • 3 ex; ibidem; 24.xii.2011; B. Kondratieff leg.; J. Háva det.; KSMA • 2 ex; Riyadh Province, Riyadh, Al Kazan, A. Aldawood leg.; *Trogoderma*; M.S. Abdel-Dayem det.; KSMA • 1 ex; ibidem; Al Shemasi; 3.x.2007; H. Aldhafer & A. Elghrbawy leg.; M.S. Abdel-Dayem det.; KSMA • 1 ex; ibidem; King Saud University, Education Farm; 25.x.2009; SW; Al Ahmari Saud leg.; J. Háva det.; KSMA • 2 ex; ibidem; 12.xi.2009; SW; Al Ahmari Saud leg.; J. Háva det.; KSMA • 1 ex; ibidem; Riyadh; vi.1988; SW (Flowers); J. Háva det.; KSMA • 2 ex; Riyadh Province, Wadi Ad Dawasir, Aly Mubark Al Baqan Farm (F81), 20°27.766'N, 44°52.024'E; Alt. 673 m; 9.v.2018; SW; Hathal et al. Leg.; J. Háva det.; KSMA • 1 ex; Tabuk Province, Tabouk, Al Madinah Rd / Desert, Aga Farm (F121), 28°28.317'N, 36°37.819'E; Alt. 750; 23.v.2018; SW; H. Aldhafer et al. Leg.; J. Háva det.; JHAC • 1 ex; ibidem; Hasan Amin Al Aly Farm (F120), 28°23.994'N, 36°44.051'E; Alt. 786; 22.v.2018; SW; H. Aldhafer et al. Leg.; J. Háva det.; KSMA • 2 ex; ibidem; J. Háva det.; JHAC • 1 ex; ibidem; 28°23.474'N, 36°51.968'E; Alt. 808 m; 14.ix.2011, LT; H. Setyaningrum & A. Al Ansi leg.; J. Háva det.; KSMA • 1 ex; ibidem; 28°25.671'N, 36°47.378'E; Alt. 797 m; 14.ix.2011, SW; H. Setyaningrum & A. Al Ansi leg.; J. Háva det.; KSMA • 1 ex; ibidem; 28°33.671'N, 36°45.356'E; Alt. 806 m; 14.ix.2011, SW; H. Setyaningrum & A. Al Ansi leg.; J. Háva det.; KSMA • 1 ex; ibidem; Aman Rd, Adnan Al Hogily Farm (F119), 28°37.676'N, 36°24.449'E; Alt. 771; 22.v.2018; SW; H. Aldhafer et al. Leg.; M.S. Abdel-Dayem det.; KSMA • 1 ex; ibidem; Duba Rd, 28°18.389'N, 36°02.874'E, Alt. 824 m; 15.ix.2011; SW; H. Setyaningrum & A. Al Ansi leg.; J. Háva det.; KSMA • 2 ex; ibidem; Duba Rd, 28°21.268'N, 36°26.654'E, Alt. 763 m; 15.ix.2011; SW; H. Setyaningrum & A. Al Ansi leg.; J. Háva det.; KSMA • 13 ex; ibidem; Duba Rd, 28°25.623'N, 36°26.602'E, Alt. 763 m; 15.ix.2011; SW; H. Setyaningrum & A. Al Ansi leg.; J. Háva det.; KSMA • 1 ex; ibidem; TADCO; 14.vi.1992; SW (Grasses); M.S. Abdel-Dayem det.; KSMA • 15 ex; without localty; J. Háva det.; KSMA.

***Attagenus posticalis* Fairmaire, 1879**

**Material examined.** SAUDI ARABIA • 1 ex; Asir Province, Al-Magardah, Wadi Khat; 31v.2012; BS; Al Ansi A. leg.; J. Háva det.; KSMA • 3 ex; ibidem; Khamis Mushiat, Wadhi Bin Hasbal, 18°35'44.3"N, 42°39'1.3"E; Alt. 1892 m; 26.iv.2011, SW; M. Sharaf, A. Al Ansi & H. Setyaningrum leg.; J. Háva det.; KSMA • 2 ex; ibidem; 18°20'01.1"N, 42°42'12.9"E; Alt. 1990 m; 27.iv.2011; SW; M. Sharaf, A. Al Ansi & H. Setyaningrum leg.; M.S. Abdel-Dayem det.; KSMA • 1 ex; Baha Province, Al Mukhwah, Shada Al Ala, 19°51.066'N, 41°18.037'E; Alt. 1325 m; 21.iv.2014; LT; Al Dhafer H., Fadl H., Abdel-Dayem M. & El Torkey A. leg.; J. Háva det.; KSMA • 1 ex; ibidem; Thee Ain Village, 19°55.774'N, 41°26.574'E; Alt. 754 m; 10.iii.2012; LT; H. Aldhafer, H. Fadl, A. El Torkey, M. Abdel-Dayem & H. Setyaningrum leg.; M.S. Abdel-Dayem det.; KSMA • 1 ex; ibidem; Thee Ain, 20 km S. Of Baha; 19°55' 54"N, 41°26' 29"E; 13.x.2010; H. Aldhafer, B. Kondratieff, H. Al Fadly, & A. Gharbawy leg.; J. Háva det.; KSMA • 1 ex; Eastern Province, Al Ahsa, Al Wozeyh, Al Riyanah Farm (F1), 25.5166667°N, 49.75477778°E; Alt. 126 m; 18.iv.2018; SW; H. Aldafer et al. leg.; M.S. Abdel-Dayem det.; KSMA • 1 ex; ibidem; VC; H. Aldafer et al. leg.; M.S. Abdel-Dayem det.; KSMA • 2 ex; Eastern Province, Qaryah Al-Ulya, Fahad Al Dobas Farm (F11), 27.5768611°N, 47.7178055°E; 20.iv.2018; LT; H. Aldafer et al. leg.; J. Háva det.; KSMA • 1 ex; Hail Province, Hail, Hail-Qasim Rd., 27°27.737'N, 41°47.233'E; Alt. 992 m; 17.ix.2011; SW; H. Setyaningrum & A. Al Ansi leg.; J. Háva det.; KSMA • 5 ex; Jazan Province, Abu Arish, Ali Abu Ashwari Farm (F87), 16°59.923'N, 42°50.059'E; Alt. 73 m; 10.v.2018; LT; H. Aldhafer et al.; J. Háva det.; KSMA • 5 ex; ibidem; Sanba Farm (F89), 17°01.218'N, 42°37.643'E; Alt. 26 m; 11.v.2018; SW; H. Aldhafer et al. leg.; J. Háva det.; KSMA • 1 ex; Jazan Province, Ahad Al Masarha, 17°02'28.0"N, 42°52'38.3"E; 11.iii.2010; LT; H. Aldhafer & A. Elghrbawy leg.; M.S. Abdel-Dayem det.; KSMA • 1 ex; Jazan Province, Al Aridah, Wadi Al Rad, 17°04.096'N, 43°04.333'E; Alt. 192 m; 21.v.2012; Al Ansi A. leg.; M.S. Abdel-Dayem det.; KSMA • 1 ex; Jazan Province, Damad, Al Shugayri, Shiekh Ahmed Famr (F95), 17°07.659'N, 42°48.748'E; Alt. 90 m; 21.v.2018; SW; H. Aldhafer et al. leg.; M.S. Abdel-Dayem det.; KSMA • 3 ex; Jazan Province, Farasan, Farasan Island, Al-Kosar; 16°40'14.8"N, 42°08'54.9"E; Alt. 7 m; 25.i.2017; SW; U.M. Abu El-Ghiet & T.M. Elsheikh leg.; M.S. Abdel-Dayem det.; KSMA • 1 ex; ibidem; Sajid Island, Khotp; 16°52'27.9"N, 41°54'43.9"E; Alt. 9 m; 6.ix.2017; SW; U.M. Abu El-Ghiet & T.M. Elsheikh leg.; M.S. Abdel-Dayem det.; KSMA • 7 ex; ibidem; Sajid Island, Sajid; 16°45'42.6"N, 41°59'56.0"E; Alt. 6 m; 25.i.2017; SW; U.M. Abu El-Ghiet & T.M. Elsheikh leg.; M.S. Abdel-Dayem det.; KSMA • 5 ex; Jazan Province, Sabya, Al Khawarah, In front of Alesayi Farm (F88), 17°07.467'N, 42°37.612'E; Alt. 36 m; 11.v.2018; SW; H. Aldhafer et al. leg.; M.S. Abdel-Dayem det.; KSMA • 3 ex; Jouf Province, Tabarjal, Basieta, Al Jouf Agric. Comp., 29°52'49.2"N, 38°35'26"E; 29.v.2022; SW (Apple); A. Al Ansi et al. leg.; J. Háva det.; KSMA • 1 ex; ibidem; SW (Plum); A. Al Ansi et al. leg.; M.S. Abdel-Dayem det.; KSMA • 2 ex; ibidem; JADCO ( F126), 30°15.598'N, 38°14.616'E; Alt. 587 m; 23.v.2018; H. Aldhafer et al. leg.; J. Háva det.; KSMA • 1 ex; ibidem; JADCO ( F128), 29°48.791'N, 38°28.604'E; Alt. 625 m; 24.v.2018; SW (Alfalfa); H. Aldhafer et al. leg.; M.S. Abdel-Dayem det.; KSMA • 3 ex; ibidem; VC (Alfalfa); H. Aldhafer et al. leg.; M.S. Abdel-Dayem det.; KSMA • 1 ex; ibidem; NADEC ( F138), 29°52.29'N, 38°18.403'E; Alt. 635 m; 24.v.2018; SW (Apricot); H. Aldhafer et al. leg.; M.S. Abdel-Dayem det.; KSMA • 3 ex; ibidem; SW (Olive); H. Aldhafer et al. leg.; M.S. Abdel-Dayem det.; KSMA • 4 ex; Jouf Province, Tabarjal, 30°31'N, 38°14'E; 27.v.2022, SW (Spinach); Al Ansi et al. leg.; J. Háva det.; KSMA • 1 ex; Madinah Province, Al-Ula, Abu Khrait, Abdullah Awad Al Balawi Farm (F104), 26°16.767'N, 37°57.738'E; Alt. 494 m; 19.v.2018; LT; H. Aldhafer et al. leg.; M.S. Abdel-Dayem det.; KSMA • 2 ex; Makka Province, Al Ardiyat, Wadi Yabah, 19°16.271'N, 41°48.464'E; Alt. 411 m; 12.iii.2012; LT; M. Abdel-Dayem & A. El Torkey leg.; J. Háva det.; KSMA • 1 ex; Makkah Province, Maysan, Taif Rd. W. Rakhamat, 20°42'N, 40°59'E; Alt. 1616 m; 4.vi.2012; BS; M.S. Abdeldayem, leg.; J. Háva det.; KSMA • 1 ex; ibidem; HP; M.S. Abdeldayem, leg.; J. Háva det.; KSMA • 1 ex; Makkah Province, Maysan, Wadi Tutubah, 20°37.103'N, 41°17.474'E, Alt. 1419 m, 26.iv.2013; A. Al Ansi & M. Al Harbi leg.; J. Háva det.; KSMA • 2 ex; Najran Province; Najran, Alshurfa, 17°31'N, 44°15'E; Alt. 1342 m; 13.iii.-30.iii.2013; MT; H.A. Dawah lgt.; J. Háva det.; NMWC • 1 ex; ibidem; J. Háva det.; JHAC • 1 ex; Qassim Province, Al Badai, Al Wadi , Mubarak bin Mohammed Al Qadeem Farm (F40), 25°47.429'N, 43°45.647'E; Alt. 711 m; 01.v.2018; SW; Hathal et al. leg.; M.S. Abdel-Dayem det.; KSMA • 1 ex; ibidem; Al Wusta, Al Suhaibani Farm (F56), 25°57.355'N, 43°47.72'E; Alt. 708 m; 01.v.2018; VC; Hathal et al. leg.; M.S. Abdel-Dayem det.; KSMA • 4 ex; Qassim Province, Al-Midhnab, Abdulaziz Al Noqaithan Farm (F47), 25°51.732'N, 44°13.845'E; Alt. 641 m; 02.v.2018; SW; Hathal et al. leg.; J. Háva det.; KSMA • 2 ex; Qassim Province, Al-Shamasiyah, Al Oufi Farm (F54), 26°15.902'N, 43°16.639'E; Alt. 664 m; 03.v.2018; SW; Hathal et al. leg.; M.S. Abdel-Dayem det.; KSMA • 2 ex; Qassim Province, Unyzah, Ar Rawghani, Mohammed Saad Al Mutairi Farm (F42), 26°08.742'N, 43°58.396'E; Alt. 664 m; 02.v.2018; VC; Hathal et al. leg.; M.S. Abdel-Dayem det.; KSMA • 1 ex; Riyadh Province, Ad Duwadimi, Sajir, Al Khudairy Farm (F34), 25°07.431'N, 44°39.277'E; Alt 693 m; 29.iv.2018; SW; Hathal et al. leg.; M.S. Abdel-Dayem det.; KSMA • 2 ex; ibidem; At Tasrir, Abu Abdullah Al Mesiar Farm (F37), 25°10.471'N, 45°32.500'E; Alt 735 m; 28.iv.2018; SW; Hathal et al. leg.; M.S. Abdel-Dayem det.; KSMA • 1 ex; Riyadh Province, Al Aflag, Al Naifiyah, Farshet Sheal, 22°24.935'N, 46°35.287'E; Alt 599 m; 07.viii.2015; PT (*Maerua crassifolia* and *Calotropis procera*); Al Dhafer H., Abdel-Dayem M., El Torkey A., El Gharbawy A. & Soliman A. leg.; M.S. Abdel-Dayem det.; KSMA • 6 ex; Riyadh Province, Al-Hariq, Al Rukiyah, Al Azbah Farm (F67), 23°36.825'N, 46°28.78'E; Alt 685 m; 19.v.2018; Hathal et al. leg.; J. Háva det.; KSMA • 1 ex; Riyadh Province, Al Kharj, Nasir Fahad Faisal Al Farhan Farm (F15), 24°13'52"N, 47°14'14"E; 16.iii.2021; SW (Lemon); Hamdah et al. leg.; M.S. Abdel-Dayem det.; KSMA • 1 ex; ibidem; Al-Shahwan Farm; 24.iii.2010; M. Al-Dossary leg.; M.S. Abdel-Dayem det.; KSMA • 4 ex; ibidem; Wadi Nesah, Hamad Al Fawa Farm (F16), 24°13.079'N, 47°0.428'E; Alt. 508 m; 24.iv.2018; SW; H. Al Dhafer et al. leg.; J. Háva det.; KSMA • 4 ex; ibidem; Yamamah, Taghlubiah Farm (F13), 24°38.257'N, 47°41.701'E; Alt. 429 m; 24.iv.2018; VC; H. Al Dhafer et al. leg.; M.S. Abdel-Dayem det.; KSMA • 1 ex; Riyadh Province, Al Quwayiyah, Rawdah Al Harmalyiah, 24.28999°N, 45.14272°E; Alt 796 m; 18.iv.2015; SW; Al Dhafer H., Abdel-Dayem M., El Torkey A., El Gharbawy A. & Soliman A. leg.; M.S. Abdel-Dayem det.; KSMA • 2 ex; ibidem; PT; Al Dhafer H., Abdel-Dayem M., El Torkey A., El Gharbawy A. & Soliman A. leg.; M.S. Abdel-Dayem det.; KSMA • 2 ex; Riyadh Province, As Sulayyil, Uruq Bani Ma´arid, W. Ghadai, Site Y, 19°20'52"N, 45°9'11.58"E; Alt. 974 m; 7.iv.2021; SW; Al Dhafer H., Soliman A. & Rassol I. leg.; J. Háva det.; KSMA • 1 ex; Riyadh Province, Diriyah; 7.ix.1990; SW; Al Motalq leg.; M.S. Abdel-Dayem det.; KSMA • 3 ex; ibidem; 4.v; SW (Coriander); M.S. Abdel-Dayem det.; KSMA • 3 ex; ibidem; 20.viii; SW; Saleh leg.; M.S. Abdel-Dayem det.; KSMA • 1 ex; ibidem;x.1989; SW (Alfalfa); M.S. Abdel-Dayem det.; KSMA • 1 ex; ibidem; Ammariyah, Animal Production Dept. Farm; 23.iii.2011; H. Setyaningrum leg.; M.S. Abdel-Dayem det.; KSMA • 3 ex; ibidem; Al Uyaynah; 28.iv.2010; SW; Al-Otaibi A. leg.; J. Háva det.; KSMA • 1 ex; ibidem; 4.v.2006; SW; Hatab Al Sebiay leg.; M.S. Abdel-Dayem det.; KSMA • 2 ex; ibidem; 12.v.2010; SW; A. Al-Hashel leg.; M.S. Abdel-Dayem det.; KSMA • 1 ex; ibidem; Wadi Hanifah, WHN04, 24.905003°N, 46.179133°E; Alt. 810.794 m; 29.iv.2015; PT; Abdel-Dayem M. et al. leg.; J. Háva det.; KSMA • 1 ex; ibidem; Wassil; 11.v.2010; SW; Mureed Husain leg.; M.S. Abdel-Dayem det.; KSMA • 1 ex; ibidem; 2.vi.2009; SW; A. Baziad leg.; M.S. Abdel-Dayem det.; KSMA • 1 ex; Riyadh Province; Howtat Bani Tamim, 23°49'35"N, 46°18'58"E; 15.iv.2010; SW (Alfalfa); Abdulah Al-Othman leg.; M.S. Abdel-Dayem det.; KSMA • 1 ex; ibidem; Hariq Road, 23°32.028'N, 46°47.330'E; Alt. 584 m; 8.v.2012; VC; Al Ansi A. leg.; M.S. Abdel-Dayem det.; KSMA • 1 ex; ibidem; Wadi Birk, F70, 23°15.839'N, 46°43.31'E; Alt. 608 m; 8.v.2012; VC; Al Ansi A. leg.; M.S. Abdel-Dayem det.; KSMA • 7 ex; Riyadh Province; Huraymila, Wadi Hurayamala; Alt. 770 m; 9.vi.1988; C.V.Mills lgt.; J. Háva det.; FSCA • 1 ex; Riyadh Province, Ramah, Rhodet Khorim, 25°25.943'N, 47°13.863'E; Alt. 572 m; 20.iv.2013; SW; M.S. Abdel-Dayem det.; KSMA • 1 ex; ibidem; 28.iv.2012; BS (*Acacia ehrenbergiana*); M.S. Abdel-Dayem det.; KSMA • 2 ex; ibidem; 14.v.2012; PT (*Calotropis procera*); J. Háva det.; KSMA • 3 ex; ibidem; 26.v.2012; BS (*Acacia gerrardii*); J. Háva det.; KSMA • 1 ex; ibidem; 26.v.2012; VC (*Acacia gerrardii*); M.S. Abdel-Dayem det.; KSMA • 1 ex; ibidem; 23.vi.2012; BS (*Acacia gerrardii*); M.S. Abdel-Dayem det.; KSMA • 8 ex; ibidem; 26.viii.2013; BS (*Ziziphus nummularia*); M.S. Abdel-Dayem det.; KSMA • 1 ex; ibidem; 25°22.986'N, 47°16.712'E; Alt. 559 m; 23.vi.2012; VC (*Ziziphus nummularia*); M.S. Abdel-Dayem det.; KSMA • 1 ex; ibidem; 13.x.2012; SW (*Rhazya steicta*); J. Háva det.; KSMA • 7 ex; ibidem; 24.ix.2012;BS (*Ziziphus nummularia*); J. Háva det.; KSMA • 1 ex; ibidem; 24.ix.2012;PT (*Rhazya stricta*); M.S. Abdel-Dayem det.; KSMA • 1 ex; ibidem; 4.xi.2012;BS (*Ziziphus nummularia*); M.S. Abdel-Dayem det.; KSMA • 1 ex; Riyadh Province, Riyadh, Dirab; 8.iv; SW (Grasses); Adel leg.; M.S. Abdel-Dayem det.; KSMA • 1 ex; Riyadh Province, Riyadh, King Saud University (KSU); 11.xi.2010 • 1 ex; ibidem; Education Farm; 15.x.2009; SW; H. Setyaningrum leg.; J. Háva det.; KSMA • 1 ex; Riyadh Province, Riyadh, Al Mousa Farm; 7.iv.2010; SW; Al-Dossary M. leg.; M.S. Abdel-Dayem det.; KSMA • 1 ex; Riyadh Province, Shagra, Al Sumit Farm (F29), 25°17.232'N, 45°11.682'E; Alt. 726 m; 28.iv.2018; SW; Hathal et al. leg.; M.S. Abdel-Dayem det.; KSMA • 1 ex; ibidem; Ushaiger, Abdulaziz Al Sunaidi Farm (F33), 25°24.512'N, 45°17.945'E; Alt. 660 m; 28.iv.2018; SW; Hathal et al. leg.; M.S. Abdel-Dayem det.; KSMA • 1 ex; Riyadh Province, Wadi Ad-Dawasir, Al Muhmadiyah Farm (F78), 25°25.497'N, 44°43.563'E; Alt. 700 m; 9.v.2018; SW; Hathal et al. leg.; J. Háva det.; KSMA • 1 ex; ibidem; VC; Hathal et al. leg.; M.S. Abdel-Dayem det.; KSMA • 1 ex; ibidem; Nasser Dehim Al Motlaqah Farm (F76), 25°26.548'N, 44°45.599'E; Alt. 693 m; 9.v.2018; SW; Hathal et al. leg.; M.S. Abdel-Dayem det.; KSMA • 1 ex; ibidem; VC; Hathal et al. leg.; M.S. Abdel-Dayem det.; KSMA • 1 ex; Tabuk Province, Duba Rd, 28°25.623'N 36°26.602'E; Alt. 763 m; 15.ix.2011; SW; H. Setyaningrum & A. Al Ansi leg.; J. Háva det.; KSMA • 22 ex; Tabuk Province, Tabouk, ASTRA (F115), 28°40.364'N, 36°19.619'E; Alt. 756 m; 22.v.2018; SW (Apricot); Hathal et al. leg.; J. Háva det.; KSMA • 2 ex; ibidem; Tabouk-Madinah Rd, Aga Farm (F121), 28°28.317'N, 36°37.819'E; Alt. 750 m; 23.v.2018; SW; Hathal et al. leg.; M.S. Abdel-Dayem det.; KSMA • 1 ex; ibidem; Al Husein Farm, 28°23.269'N, 36°43.269'E; Alt. 799 m; 14.ix.2011; SW; H. Setyaningrum & A. Al Ansi leg.; M.S. Abdel-Dayem det.; KSMA • 5 ex; ibidem; F123, 28°3.796'N, 36°45.406'E; Alt. 774 m; 22.v.2018; SW; Hathal et al. leg.; J. Háva det.; KSMA • 1 ex; ibidem; 28°23.474'N, 36°51.968'E; Alt. 808 m; 14.ix.2011; LT; H. Setyaningrum & A. Al Ansi leg.; M.S. Abdel-Dayem det.; KSMA • 1 ex; ibidem; SW; H. Setyaningrum & A. Al Ansi leg.; M.S. Abdel-Dayem det.; KSMA • 1 ex; ibidem; 28°25.623'N, 36°26.602'E; Alt. 763 m; 14.ix.2011; SW; H. Setyaningrum & A. Al Ansi leg.; M.S. Abdel-Dayem det.; KSMA • 1 ex; ibidem; 28°33.671'N, 36°45.356'E; Alt. 806 m; 14.ix.2011; SW; H. Setyaningrum & A. Al Ansi leg.; M.S. Abdel-Dayem det.; KSMA • 19 ex; ibidem; Uyanah, Abdullah khader Al Atawi Farm (F118), 28°53.209'N, 36°07.201'E; Alt. 733 m; 14.ix.2011; SW; Hathal et al. leg.; M.S. Abdel-Dayem det.; KSMA • 17 ex; without data; M.S. Abdel-Dayem det.; KSMA.

***Attagenus vanharteni* Háva, 2009**

**Material examined.** SAUDI ARABIA • 1 ex; Jouf, Sakaka, Abdullah Ibrahim Farm, 30°00'9.6"N, 40°01'58.3"E; 21.vi.2022; SW (Alfalfa); Al Ansi et al. leg.; M.S. Abdel-Dayem det.; KSMA • 1 ♀; Jouf, Tabarjal, Basita, Mubarak Aldwsari Farm, 30°08'59.7"N, 38°11'50"E; 30.vi.2022; SW (Parsely); Al Ansi et al. leg.; J. Háva det.; KSMA • 2 ex; Madinah Province, Al-Ula, Abu Khrait, Abdullah Awad Al Balawi Farm (F104), 26°16.767'N, 37°57.738'E; Alt. 494 m; 19.v.2018; LT; Hathal et al. leg.; J: Háva det.; KSMA • 2 ex; Najran Province, Yadamah, Uruq Bani Ma´arid, W. Sudar, Site CX, 19.19290°N, 45.13448°E; Alt. 980 m; 2.vi.2021; LT; Abdel-Dayem M., Soliman A. & Sonbati S. leg.; J. Háva det.; KSMA • 1 ex; ibidem; JHAC • 1 ex; ibidem; Site XY, 19°11'36"N, 45°08'13"E; Alt. 947 m; 8-9.iv.2021; PT; Al Dhafer H., Soliman A. & Rassol I. leg.; M.S. Abdel-Dayem det.; KSMA • 2 ex; ibidem; SW; Al Dhafer H., Soliman A. & Rassol I. leg.;M.S. Abdel-Dayem det.; KSMA • 1 ♀; ibidem; Site MC-E, 19.14764°N, 45.24627°E; Alt. 927 m; 2.vi.2021; LT; Abdel-Dayem M., Soliman A. & Sonbati S. leg.; J. Háva det.; KSMA • 3 ex; Riyadh Province, Al Aflag, Al Naifiyah, Farshet Sheaal, 22.40318°N 46.59209°E; Alt. 596 m; 13.iv.2015; PT (*Rhazya stricta*); Al Dhafer H., Abdel-Dayem M., El Torkey A., El Gharbawy A. & Soliman A. leg.; M.S. Abdel-Dayem det.; KSMA • 1 ex; ibidem; 22°24.161'N 46°35.547'E; Alt. 588 m; 7.viii.2015; PT (*Rhazya stricta*); Al Dhafer H., Abdel-Dayem M., El Torkey A., El Gharbawy A. & Soliman A. leg.; J. Háva det.; KSMA • 6 ex; ibidem; 22.40962°N, 46.59249°E; Alt. 601 m; 12.iv.2015; LT; Abdel-Dayem M. et al. leg.; M.S. Abdel-Dayem det.; KSMA • 8 ex; ibidem; 13.iv.2015; PT (*Leptadenia pyrotechnica*); Al Dhafer H., Abdel-Dayem M., El Torkey A., El Gharbawy A. & Soliman A. leg.; M.S. Abdel-Dayem det.; KSMA • 1 ex; ibidem; J. Háva det.; JHAC • 8 ex; ibidem; 22.41559°N 46.58806°E; Alt. 602 m; 13.iv.2015; PT (*Maerua crassifolia* & *Calotropis procera*); Al Dhafer H., Abdel-Dayem M., El Torkey A., El Gharbawy A. & Soliman A. leg.; M.S. Abdel-Dayem det.; KSMA • 11 ex; ibidem; 22.4279°N, 46.5747°E; Alt. 612 m; 13.iv.2015; PT (*Acacia*); Al Dhafer H., Abdel-Dayem M., El Torkey A., El Gharbawy A. & Soliman A. leg.; J. Háva det.; KSMA • 3 ex; ibidem; 22°23.475'N, 46°35.045'E; Alt. 592 m; 15.x.2015; LT; Al Dhafer H., Abdel-Dayem M., El Torkey A., El Gharbawy A. & Soliman A. leg.; J. Háva det.; KSMA • 3 ex; Riyadh Province, Al Quwayiyah, Rawdha Al Harmalyiah, 24.30964°N, 45.16656°E; Alt. 774 m; PT (Alfalfa); Al Dhafer H., Abdel-Dayem M., El Torkey A., El Gharbawy A. & Soliman A. leg.; J. Háva det.; KSMA • 1 ex; ibidem; 24.30619°N, 45.17958°E; Alt. 769 m; 20.iv.2015; PT4; Al Dhafer H., Abdel-Dayem M., El Torkey A., El Gharbawy A. & Soliman A. leg.; J. Háva det.; KSMA • 2 ex; ibidem; 24.33714°N, 45.15429°E; Alt. 774 m; 20.iv.2015; PT; Al Dhafer H., Abdel-Dayem M., El Torkey A., El Gharbawy A. & Soliman A. leg.; M.S..Abdel-Dayem det.; KSMA • 3 ex; ibidem; 27.x.2015; LT; Al Dhafer H., Abdel-Dayem M., El Torkey A., El Gharbawy A. & Soliman A. leg.; J. Háva det.; KSMA • 2 ex; Riyadh Province, As Sulayyil, Uruq Bani Ma´arid, W. Ghadai, Site Y, 19°20'52N, 45°9'11.58"E; Alt. 974 m; 7.iv.2021; SW; Al Dhafer H., Soliman A. & Rassol I. leg.; M.S. Abdel-Dayem det.; KSMA • 1 ex; Riyadh Province, Az Zulfi, Rawdet Al Sabalh, 26°21.228'N, 44°58.999'E; Alt. 678 m; 26.viii.2015; LT; Al Dhafer H., Fadl H., Abdel-Dayem M., El Gharbawy A. El Torkey A. & Soliman A. leg.; J. Háva det.; KSMA • 1 ex; ibidem; 25.x.2015; LT; M.S. Abdel-Dayem et al. leg.; M.S. Abdel-Dayemdet.; KSMA • 1 ex; ibidem; 26°21.524'N, 44°59.101'E; Alt. 669 m; 20.v.2015; PT; M.S. Abdel-Dayem et al. leg.; M.S. Abdel-Dayemdet.; KSMA • 3 ex; ibidem; 26°22.040'N, 44°59.137'E; Alt. 670 m; 20.v.2015; PT; M.S. Abdel-Dayem et al. leg.; M.S. Abdel-Dayemdet.; KSMA • 1 ex; ibidem; 26°22.289'N, 44°58.646'E; Alt. 661 m; 20.v.2015; LT; M.S. Abdel-Dayem et al. leg.; M.S. Abdel-Dayemdet.; KSMA • 3 ex; ibidem; 26°22.479'N, 44°58.241'E; Alt. 670 m; 20.v.2015; PT; M.S. Abdel-Dayem et al. leg.; M.S. Abdel-Dayemd et.; KSMA • 1 ex; Riyadh Province, Dhurma, Soultan Ibrahim Ad-dhidah Farm, 24.612777°N, 46.12972222°E; Alt. 625 m; 20.iv.2022; SW (Arugula); Chebbi H. & Abbad H. leg.; M.S. Abdel-Dayemd et.; KSMA • 1 ex; Riyadh Province, Diriyah, Al Uyaynah, 10.iii.2010; SW; Al-Salem B. leg.; M.S. Abdel-Dayem det.; KSMA • 2 ex; ibidem; Wadi Hanifah, WHN01, 24.9055°N, 46.188775°E; Alt. 814.141 m; 29.iv.2015; PT; Abdel-Dayem M. et al. leg.; J. Háva det.; KSMA • 1 ex; ibidem; 12.x.2015; PT; Abdel-Dayem M. et al. leg.; J. Háva det.; KSMA • 1 ex; ibidem; WHN02, 24.9119°N, 46.187792°E; Alt. 806.755 m; 29.iv.2015; PT; Abdel-Dayem M. et al. leg.; J. Háva det.; KSMA • 1 ex; ibidem; 12.x.2015; PT; Abdel-Dayem M. et al. leg.; J. Háva det.; KSMA • 1 ex; ibidem; WHN03, 24.90702°N, 46.181702°E; Alt. 809.224 m; 29.iv.2015, PT; Abdel-Dayem M. et al. leg.; J. Háva det.; KSMA • 1 ex; ibidem; 12.x.2015; PT; Abdel-Dayem M. et al. leg.; J. Háva det.; KSMA • 2 ex; ibidem; J. Háva det.; JHAC • 2 ex; ibidem; WHS02, 24.86891°N, 46.457495°E; Alt. 698.912 m; 29.iv.2015, PT; Abdel-Dayem M. et al. leg.; J. Háva det.; KSMA • 1 ex; ibidem; 27.viii.2015; PT; Abdel-Dayem M. et al. leg.; J. Háva det.; KSMA • 3 ex; ibidem; 12.x.2015; PT; Abdel-Dayem M. et al. leg.; J. Háva det.; KSMA • 1 ex; ibidem; WHS05, 24.86474°N, 46.461243°E; Alt. 693.76 m; 29.iv.2015, PT; Abdel-Dayem M. et al. leg.; J. Háva det.; KSMA • 4 ex; ibidem; 12.x.2015; PT; Abdel-Dayem M. et al. leg.; J. Háva det.; KSMA • 2 ♂; Riyadh Province, Hotat Bani Tamim, Wadi Birk, Farm 2 (F70), 23°15.839'N, 46°43.31'E; Alt. 608 m; 5.v.2018, LT, Hathal et al. leg.; J. Háva det.; KSMA • 3 ex; ibidem; 8.v.2018, LT, Hathal et al. leg.; J. Háva det.; KSMA • 3 ex; Riyadh Province, Huraymila, Wadi Hurayamala; Alt. 770 m; 9.vi.1988; C.V.Mills lgt.; J. Háva det.; FSCA • 1 ex; ibidem; J. Háva det.; JHAC • 2 ex; Riyadh Province, Ramah, Rhodet Khorim, 25°22.986'N, 47°16.712'E; Alt. 559 m; 2.vi.2013; PT (*Acacia ehrenbergiana*); J. Háva det.; KSMA • 1 ♀; ibidem; 25°25.943'N, 47°13.863'E; Alt. 572 m; 14.v.2012; PT (*Calotropis procera*); *Attagenus lobatus*; J. Háva det.; KSMA • 3 ex; ibidem; M.S. Abdel-Dayem det.; KSMA • 1 ex; ibidem; 26.v.2012; SW (*Rhazya stricta*); J. Háva det.; KSMA • 1 ex; ibidem; BS (*Acacia gerrardii*); M.S. Abdel-Dayem det.; KSMA • 5 ex; ibidem; SW; J. Háva det.; KSMA • 1 ex; ibidem; 27.v.2012; MT; M.S. Abdel-Dayem det.; KSMA • 1 ex; ibidem; 9.vi.2012; VC (*Calotropis procera*); M.S. Abdel-Dayem det.; KSMA • 2 ex; ibidem; 30.vi.2012; PT (*Acacia gerrardii*); J. Háva det.; KSMA • 1 ex; ibidem; 7.viii.2013; SW (*Rhazya stricta*); J. Háva det.; KSMA • 1 ex; ibidem; 27.viii.2012; PT (*Acacia gerrardii*); J. Háva det.; KSMA • 1 ex; ibidem;9.ix.2012; SW (*Rhazya stricta*); M.S. Abdel-Dayem det.; KSMA • 1 ex; ibidem;24.ix.2012; SW (*Acacia gerrardii*); M.S. Abdel-Dayem det.; KSMA • 1 ex; Tabouk Province, Tabuk, Duba Rd, 28°21.268'N, 36°26.654'E; Alt.783 m; 15.ix.2011; SW; H. Setyaningrum & A. Al Ansi leg.; J. Háva det.; KSMA.
